# Next-Generation Breeding Strategies for Climate-Ready Crops

**DOI:** 10.3389/fpls.2021.620420

**Published:** 2021-07-21

**Authors:** Ali Razzaq, Parwinder Kaur, Naheed Akhter, Shabir Hussain Wani, Fozia Saleem

**Affiliations:** ^1^Centre of Agricultural Biochemistry and Biotechnology (CABB), University of Agriculture, Faisalabad, Pakistan; ^2^UWA School of Agriculture and Environment, The University of Western Australia, Perth, WA, Australia; ^3^College of Allied Health Professional, Faculty of Medical Sciences, Government College University Faisalabad, Faisalabad, Pakistan; ^4^Mountain Research Center for Field Crops, Khudwani, Sher-e-Kashmir University of Agricultural Sciences and Technology of Kashmir, Srinagar, India

**Keywords:** food security, climate change, next-generation breeding, genomics, genome editing, CRISPR/Cas, abiotic stress, crop improvement

## Abstract

Climate change is a threat to global food security due to the reduction of crop productivity around the globe. Food security is a matter of concern for stakeholders and policymakers as the global population is predicted to bypass 10 billion in the coming years. Crop improvement *via* modern breeding techniques along with efficient agronomic practices innovations in microbiome applications, and exploiting the natural variations in underutilized crops is an excellent way forward to fulfill future food requirements. In this review, we describe the next-generation breeding tools that can be used to increase crop production by developing climate-resilient superior genotypes to cope with the future challenges of global food security. Recent innovations in genomic-assisted breeding (GAB) strategies allow the construction of highly annotated crop pan-genomes to give a snapshot of the full landscape of genetic diversity (GD) and recapture the lost gene repertoire of a species. Pan-genomes provide new platforms to exploit these unique genes or genetic variation for optimizing breeding programs. The advent of next-generation clustered regularly interspaced short palindromic repeat/CRISPR-associated (CRISPR/Cas) systems, such as prime editing, base editing, and *de nova* domestication, has institutionalized the idea that genome editing is revamped for crop improvement. Also, the availability of versatile Cas orthologs, including Cas9, Cas12, Cas13, and Cas14, improved the editing efficiency. Now, the CRISPR/Cas systems have numerous applications in crop research and successfully edit the major crop to develop resistance against abiotic and biotic stress. By adopting high-throughput phenotyping approaches and big data analytics tools like artificial intelligence (AI) and machine learning (ML), agriculture is heading toward automation or digitalization. The integration of speed breeding with genomic and phenomic tools can allow rapid gene identifications and ultimately accelerate crop improvement programs. In addition, the integration of next-generation multidisciplinary breeding platforms can open exciting avenues to develop climate-ready crops toward global food security.

## Global Scenario of Climate Changes and Food Security

**Figure 1:**
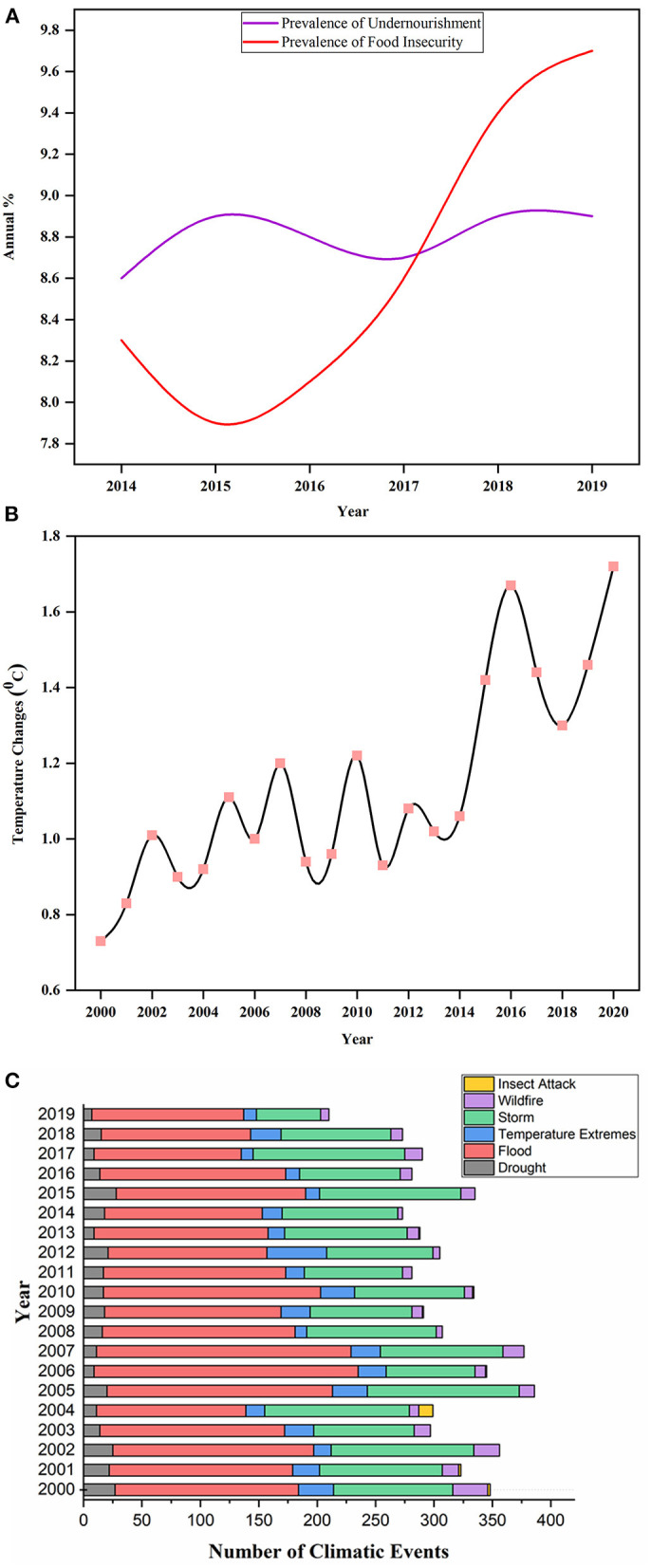
Displaying the annual prevalence of undernourishment and food insecurity percentage during 2014–2019 in **(A)**. Source: Food and Agriculture Organization (FAO) (http://www.fao.org/faostat/en/#data/FS/visualize). Illustration of changing trends in the world's temperature annually from 2000 to 2020 in **(B)**. Source: FAO (http://www.fao.org/home/en/). **(C)** depicted the total number of climatic events that occurred from 2000 to 2020 around the world. The climatic events include drought, extreme temperature, flood, storm, wildfire, and insect attack. Source: Emergency Disaster Database^2^.

Food security is the biggest challenge in feeding the continuous up-surging population. The dream of a world without hunger is only possible if agricultural productivity increases in a sustainable manner (Tilman et al., 2011). About two billion people are facing extreme micronutrient deficiencies, and over 815 million are suffering from chronic hunger. Recent evidence has revealed the increased number of undernourished people (Figure 1A) in the developing countries of Western Asia and Africa since 2014 (FAO, IFAD, UNICEF, WFP, and WHO, 2018). The situation is more deteriorated by the outbreak of severe acute respiratory syndrome coronavirus 2 (SARS-CoV-2), which severely affects the food supply chain, and its impact on global food security is yet to be understood. It is forecasted that this food crisis will upsurge people with acute hunger from 135 to 265 million by 2020 (Henry, 2020). The availability of food largely relies on economic growth, which is still crucial in dealing with both hidden and chronic hunger and poverty in underdeveloped and developing countries (Gödecke et al., 2018). Still, it might not be adequate to lessen malnutrition and hunger as there are many factors affecting global food security.

The two big challenging factors to food security are climate changes and population growth. The world's population is growing rapidly, which increases the food demand and exerts more pressure on agricultural land and other resources (Abberton et al., 2016). It is projected that the population will cross eight billion mark at the end of 2030 and is anticipated to exceed 9.7 billion before the end of 2050 (Ray et al., 2013). Agricultural productivity needs to boost to meet 49% more food requirements by 2050 to avoid extreme hunger fears^1^. An annual increase of 1.1–1.3% of the major cereal crops from the current pace is required to tackle the hunger and severe food shortage by 2050 (Fischer et al., 2014). The failure to enhance crop yield will badly affect the developing countries and can lead to famines and social discomfort. Also, there are many factors, like economic, agronomic, societal, and climatic factors, which adversely hampered crop productivity. It is a huge task for plant breeders and policymakers to cope with this massive assignment of global food safety and security (Ray et al., 2013).

Climate change is a leading aspect threatening agricultural yield worldwide. However, it is impossible to envisage the exact costs of damages caused by climate changes, in a broader perspective the crop yields will be greatly reduced. Climate change elevates the global temperature (Figure 1B) that changes geographical orders of rainfall and instigates greater concerns to agricultural production. These changes cause global warming, and an increased CO_2_ level is anticipated to affect the nutritive quality of numerous cultivars while many varieties may become unsafe because of chemical alterations in the cells. The extreme events of climate change increase the loss of agricultural land and accelerate biotic and abiotic stresses (Raza et al., 2019). The future projection and impacts of climate changes are uncertain (Allen et al., 2019), which lead to crop adaptability under diverse range of climate stress, i.e., a tough breeding goal. The dilemma becomes even bigger because the fluctuations in annual rainfall and temperature negatively affect crop growth and encourage the attacks of crop pathogens (Heeb et al., 2019). It was reported that 10–25% yield of major staple crops, including wheat, maize and rice, was reduced due to per degree rise in temperature (Deutsch et al., 2018). This is due to an increase in temperature accelerating the metabolic activities of insects and enhancing insect's food consumption rate. Likewise, the elevated level of CO_2_ makes soybean more susceptible to insect pathogens (Zavala et al., 2008). The major stress events that occurred due to climate change in the last two decades are illustrated in Figure 1C.

**Figure 2:**
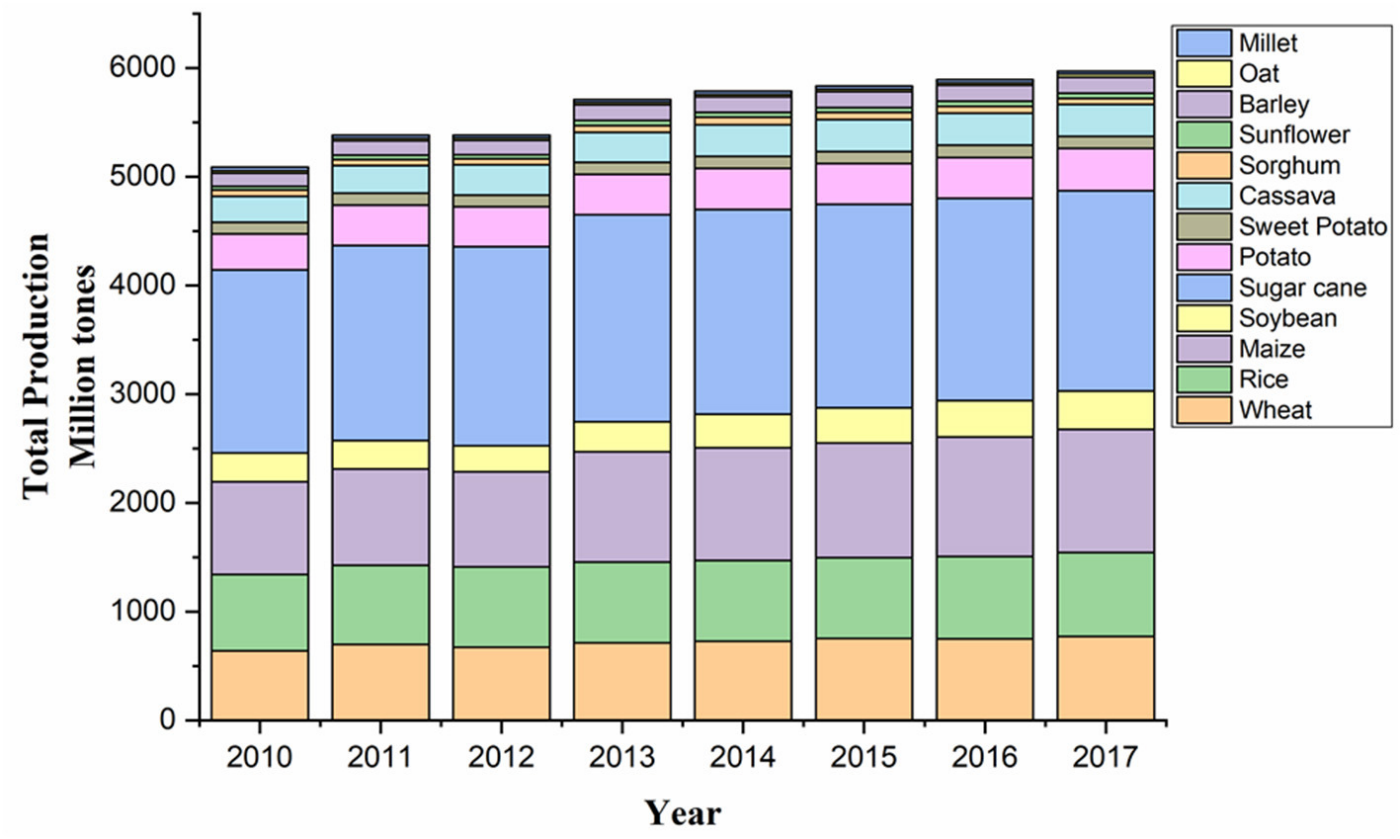
Graphical representation of the total production of major crops in the world (2010–2017). Source: Data retrieved from FAOSTAT^1^.

The reported rise in productivity of major staple crops, such as wheat, rice, maize, and soy, sharing two-thirds of total calories consumed by people is very less, although there has been a significant increase in the crop yield in recent years (Figure 2; FAO, 2017; Kim et al., 2019a). Meanwhile, the actual increase in population is much quicker than forecasted before, with the updated figure being over 9.9 billion by 2050 (Change, 2020). These current climate and population growth trends are supposed to further hamper the crop yield, thus extending the gap between food demand and food production. These challenges will greatly decide the future scenario of global food security.

A novel agricultural model, including the integrated systems of modern breeding, different agronomic practices, and plant microbiome analysis, is needed. Microbiomes or plant-associated microbes can offer crucial ecosystem facilities, which can boost crop growth and help to mitigate abiotic stresses and pathogen attacks (Arif et al., 2020). Climate-smart agriculture is gaining interest to develop climate-resilient crop varieties by adopting the next-generation breeding approaches that can withstand multidimensional stresses, including salinity, waterlogging, heat, cold, drought, and insect-pests attack.

In the present review, we argue that climate change and the unchecked growing population may reverse the progress achieved at present toward the sustainability goal of zero hunger. The current pace of crop production is insufficient to meet future demands. We highlight significant breakthrough in the plant breeding history. We advocate the next-generation breeding as a reasonably practical way forward to mitigate the impacts of climate changes and develop climate-ready crops for better resilience and improved yield. Indeed, there is a need for a comprehensive strategy, including an integrated multidisciplinary strategy (seed production, pathology, agronomy, post-harvest approaches, agriculture extension, and different breeding practices); still breeding is an obvious point for moving ahead. Hence, we discuss the fascinating technologies of genomic-assisted breeding (GAB) with a focus on advances in crop pan-genome assemblies and their application for crop improvement. We pay specific attention to cutting-edge genome editing tools like clustered regularly interspaced short palindromic repeat/CRISPR-associated (CRISPR/Cas) systems and spotlight their expanding toolbox. Furthermore, we discuss plant phenomics and their major bottlenecks that need to be overcome to bridge the gap between genomics and phenomics and focus on high-throughput phenotyping for crop improvement. We also highlight the next-generation phenomics approaches, including artificial intelligence (AI) and machine learning (ML), that can revolutionize the digital agriculture. Finally, we describe speed breeding and propose the integration of next-generation breeding technologies to expedite the crop production for food security.

## Plant Breeding the Savior

**Figure 3:**
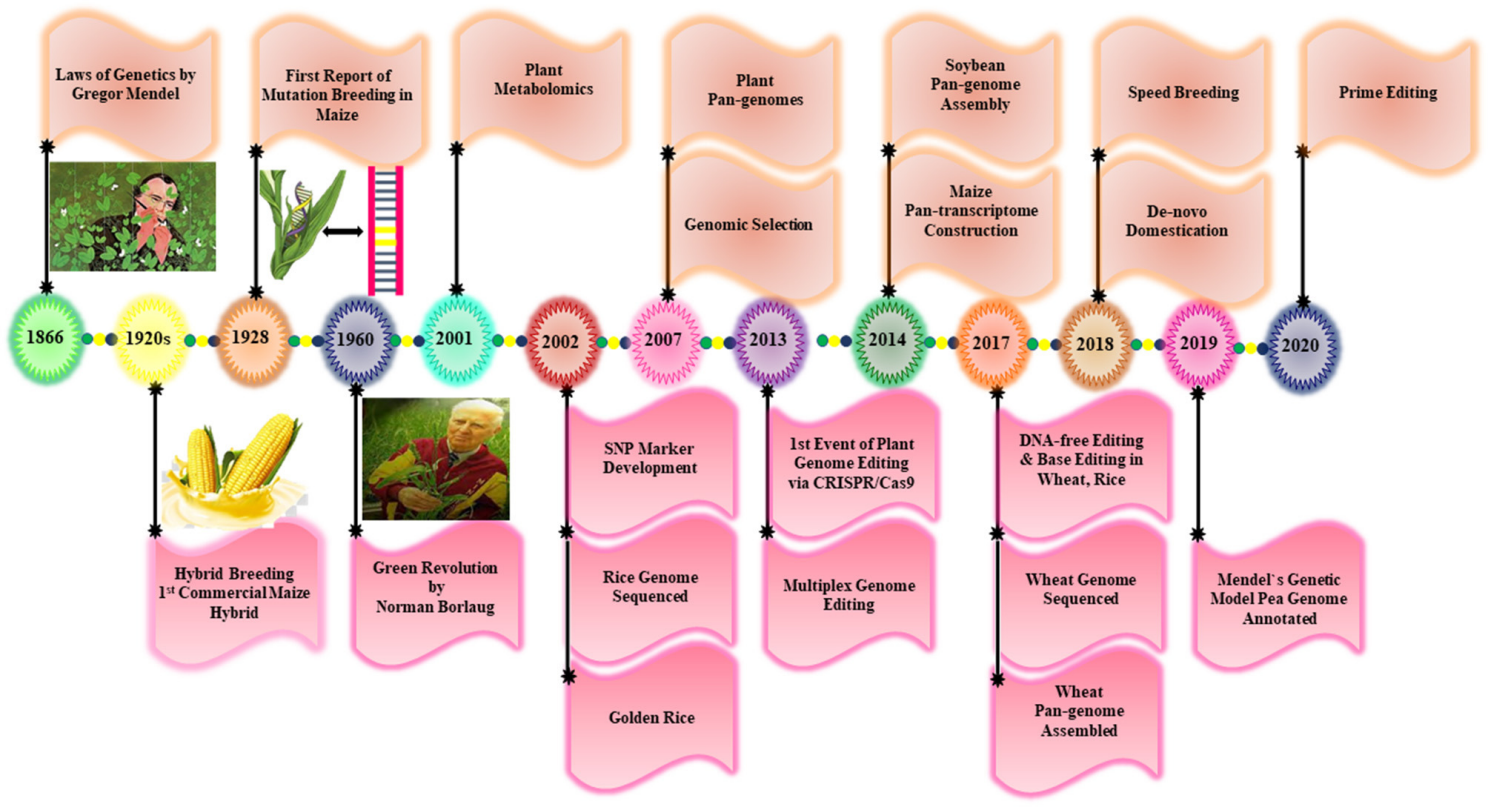
Representations of the key milestones achieved by conventional and modern plant breeding.

Approximately 10,000 years ago, plant breeding emerged as a central approach for plant domestication by exploiting wild relatives to select the desired traits through a continuous selection process over several generations for crop improvement (Purugganan and Fuller, 2009). Many important crops are cultivated extensively all around the world, which have been developed through a breeding process. Some of the major milestones achieved in the plant breeding history are spotlighted in Figure 3. For example, in the pre-genetic era, many agronomic traits were incorporated blindly into different crop species (Purugganan and Fuller, 2009). The discovery of Mendel laws of inheritance and the continuous detection of genetic elements provide new insight into plant breeding. After 100 years of constant research, the scientists are allowed to identify the genomic regions, which are named later as genes that regulate the agronomic traits in plants (McCouch et al., 2013). In the 1960's, the Green Revolution remarkably increased the yield potential of some major crops, including rice and wheat, to meet the growing food demands (Pingali, 2012). Although it has brought an enormous benefit for agriculture and humanity but also a lot of negative environmental consequences because of the unchecked application of synthetic fertilizers and pesticides. Additionally, the Green Revolution encouraged intensive breeding that resulted in the reduction of genetic diversity (GD) and the loss of several unique genes. This led to an increased attack of various insect-pests pathogens and the reduction of plant vigor to withstand extreme heat, drought salinity, and flooding conditions (Tilman et al., 2002). However, plant breeding has been under immense pressure after this period to maintain constant agricultural yield with limited resources of water, land, and fertilizers. To tackle these problems, plant scientists need to elucidate the unique genetic resources to produce superior cultivars having better stress resilience and increased grain yield.

Generally, in classical breeding, elite crop varieties have been selected *via* hybridization and a continuous screening process (Purugganan and Fuller, 2009). The fundamental features of basic plant breeding lead to the incorporation of GD through intercrossing of the plants with novel agronomic features with wild relatives or crop landraces and select the best genotypes having outstanding characters (Lavarenne et al., 2018). Investigations of wild populations can offer greater GD to introduce the desired traits to develop new crop varieties, which provide an excellent roadmap for crop improvement. For example, the introduction of diverse genetic recombination through hybridization among species may offer an exceptional chance to combat climate stresses (Becker et al., 2013). However, conventional breeding strategies for crop improvement are of limited use because of genetic drag, genetic erosion, hybridization bottlenecks, and laborious selection process (Abberton et al., 2016). It could take 10–20 years to develop a crop variety with desired traits, which make it a complicated and time-consuming endeavor (Fischer et al., 2014). On the other hand, modern breeding approaches like genetic engineering to produce genetically modified (GM) crops gained a significant progress in the last three decades to overcome the hurdles posed by conventional breeding techniques. For example, in 1994, “Flavr Savr” transgenic tomato having an improved shelf life has been approved by Food and Drug Administration (FDA) in the USA. After that, a series of GM crops like glyphosate-resistant soybeans, *Bt* cotton, *Bt* maize, and *Bt* Potato, etc., have been approved for commercial use in the USA (James, 1997). A total of 529 transgenic events reported in 33 crops have been commercialized (ISAAA database, 2021). About 91% of the total area coverage under GM crops are offered by USA, Canada, Brazil, Argentina, and India. USA is the largest grower of GM crops in the world and cultivates different improved varieties of maize (43), soybean (25), potato (43) cotton (32), and canola (22). Asian countries, such as China (canola, cotton, maize, and soybean), India (soybean and cotton), Pakistan (maize and cotton), and Bangladesh (*Bt* Brinjal), grow only a considerable number of GM crops on a limited area (ISAAA database, 2021). In spite of all these achievements, there are numerous serious concerns associated with GM crops, such as biosafety for human consumption, resistance breakdown in several pathogens, hazardous impacts on nontarget organisms, and the cost of commercialization. Also, all the European countries banned all kinds of GM organisms in their country, which make this a controversial technique for crop improvement. Owing to public safety concerns and unwillingness to accept GM crops, we need alternative next-generation breeding technologies to increase the genetic gains in plant breeding and develop intelligent models to bridge the gap between genotype and phenotype for the next-generation crop improvement.

## Fast-Forward Gab

Modern plant breeding approaches have revolutionized plant breeding and are emerged as a powerful alternative to conventional breeding. Plant genomics is extremely vital to accelerate breeding programs and crucial to improve crop performance, including trait identification and the discovery of genetic variations within the crop genome, that regulate crop performance and increase stress resilience (Bevan et al., 2017). Plant genomics is an important player of omics science that deals with the whole plant genomes for determining the structure and assessing the function of genomes. It is important to draw the information inside the genome by defining a specific arrangement of DNA sequences, which help to probe the genomic evolution and interpret molecular phylogenetic relationships (Varshney, 2016). It also assists in elucidating the function of genes and their interaction that govern the plant growth and functions under diverse agroclimatic conditions (Unamba et al., 2015).

Fast-forward genotyping and phenotyping platforms have facilitated multitrait association studies *via* genome-wide association studies (GWAS) to accurately explore the genetic make-up of crop traits. Quantitative trait loci (QTL) analysis through mapping agronomically important traits enabled to dissect the relationship between genotype and phenotype. Gene cloning and characterization, haplotype-based breeding, allele mining for stress tolerance, and tapping natural variations have open new avenues toward GAB (Leng et al., 2017). In the coming year, large data sets of genomics can offer wealth of information about the plant genomes, and GAB would become an instrumental practice to accelerate breeding to design future crops (Varshney et al., 2021).

Exclusive understanding of plant genomes is essential for next-generation sequencing- (NGS-) based trait mapping that has allowed rapid gene identification. Current advances in genome sequencing techniques have transformed plant breeding by providing access to huge plant genomes and open a new era of genomics (Bassi et al., 2016). The advanced NGS techniques enable to explore the diverse and multidimensional spectrum of genetic variations that can be linked to elucidate complex phenotypes (Unamba et al., 2015).

There are numerous genomic markers that can be utilized for the identification of plant genes through positional cloning (Bassi et al., 2016). For example, single-nucleotide polymorphism (SNP) cost-efficient, accurate, and ubiquitous in crop genomes and has been used on a large scale to screen thousands of crop germplasm (Voss-Fels and Snowdon, 2016). Although SNPs are the chief molecular markers to study the phenotypic and GD among different crop breeds, but they can also be very crucial for probing a wide range of variations linked with some important stress tolerance and agronomic characters (Saxena et al., 2014). Sequencing techniques can capture SNP accurately but are unable to generate long reads sufficient to represent the diversity. To overcome these issues, optical mapping can be executed for generating long read maps having a greater variation and complex regions (Golicz et al., 2016a). Crops like wheat and maize have larger genomes, genotyping by sequencing (GBS) coupled with NGS have been widely used for multiplexed sample sequencing (Arthur and Bennetzen, 2018). Fixed SNP genotyping arrays can be used instead of NGS-based techniques because they are economical and precise, and require less data analysis. Recently, some genotyping platforms and crop breeding chips have been complied, which could be used in GAB (Rasheed et al., 2017).

A comprehensive knowledge of genes and their regulating pathways that control the dynamic traits, such as quality, yield, and stress tolerance, would increase our knowledge to develop next-generation crop varieties. However, the less availability of genomic information and lack of significant understanding are not completely accredited to the inadequate genomic innovations but also depend on the environment–genotype interactions and phenotypic pitfalls (van Bezouw et al., 2019). In the last two decades, a significant development has been achieved in the genomic technologies that accelerate breeding programs (Varshney et al., 2017). Advanced multidisciplinary breeding platforms are required to mitigate the climate changes and accelerate the genetic gains for achieving the target demands of crop yield (Bevan et al., 2017). Upon the integration of fast-forward genomic technologies, such as genomic selection (GS), NGS, SNP-mediated marker-assisted selection, and GWAS, together with holistic phenotyping and more sophisticated bioinformatics, data analysis, and decision support tools (Varshney et al., 2018) can drive the next-generation breeding.

## The Pan-Genomes and In-Depth Exploration of Natural Variation

Crop domestication and natural evolution have severe impacts on crop genomes resulting in the impairment of major loci regulating important agronomic traits and ultimately reduce GD (Warschefsky et al., 2014). Also, the selection of superior lines for abiotic/biotic stress tolerance to improve crop yield has worsened the condition, directing to the elimination of novel stress-tolerant genes that were abundantly present in the gene pool of crop wild relatives (CWRs; Brozynska et al., 2016). Due to the reduction in GD, the crops are now more vulnerable to adverse climatic stresses, and there is an urgency to transfer all the intentions toward CWRs that contain a rich-gene pool of stress-responsive genes (McCouch et al., 2013).

Although the principal focus on studying GD is carried out by detecting the structural variations (SVs) within the genome by employing SNPs, the SVs has been considered as a principal source of GD and may include presence/absence variants (PAVs), copy number variants (CNVs), and some other diverse variations such as chromosomal translocation, inversion, and transversions (Wang et al., 2015). CNVs occur in the distinctive form of copies among individuals while PAVs occur in one genome and are absent in another (Saxena et al., 2014). PAVs and CNVs are commonly present within species in the plant genomes and play a key part to understand plant genetics (Hirsch et al., 2014). A few studies showed that the genetic variations that are mainly studied *via* SNPs/InDels are insufficient to depict the whole genomic pool of a species (Saxena et al., 2014). Furthermore, the re-sequencing technologies are centered only on a single-reference genome that has inadequate capability to detect the entire spectrum of large SVs, like CNVs and PAVs, and thus providing insufficient information for crop GD (Tao et al., 2019).

Hence, there is a need to develop multireference genomes to investigate the genome composition of all individuals, including cultivated, landraces, or wild progenitors. With the advancement in NGS technologies, a huge wave of pan-genomic studies have been launched, which open a new roadmap to determine crop evolution and adaptation across the genus, and study deep insights into genome functions with the possible application for crop breeding (Hirsch et al., 2014; Zhao et al., 2018a). Pan-genomics provides an excellent platform to study the GD, compares multiple genomes simultaneously, and recaptures the whole genetic repertoire of a species (Zhao et al., 2018a).

### Crop Pan-Genomes

The term pan-genome was firstly opted to describe the bacterial genome, and it depicts the whole gene stock of different individuals present in a species. Pan-genomes are comprised of two regions, including the core genome that represents the core genes present in all individuals and dispensable genome consisting of accessory or variable genes, particularly shared by few group members and missing in remaining individuals (Tettelin et al., 2005). The core genes tend to be conserved and perform important functions while the dispensable genome provides a greater GD and is supposed to carry the stress-responsive genes in a species. In addition, the dispensable genome is a key component to regulate the phenotypic variation related to agronomic traits, and is crucial for increasing agricultural productivity. It is also a major contributor to the domestication and adaptive evolution of species (Tranchant-Dubreuil et al., 2018; Jayakodi et al., 2021). In recent years, pan-genomes concepts have moved toward crop plant research, and pan-genomes of some major crops have been constructed (Hurgobin et al., 2018).

The resources of crop pan-genomes rather than single-reference genomes will accelerate molecular breeding and provide a multidimensional understanding of genomic variations (Golicz et al., 2016a,b). SVs and PAVs are vital to understanding the variable genes, which have been revealed to control the genes related to stress, like biotic stress, tolerance in soybean (McHale et al., 2012), *Brassica oleracea* (Bayer et al., 2019), muskmelon (González et al., 2013), and phosphor uptake capacity of rice (Schatz et al., 2014). In general, pan-genomes explore new trends to study GD, which will play a key part in probing genomic variations and offer a speedy approach to scanning complex gene sets for crop improvement (Tao et al., 2019).

Pan-genomes of rice (Schatz et al., 2014; Zhao et al., 2018a), tomato (Gao et al., 2019), soybean (Li et al., 2014; Liu et al., 2020b), sunflower (Hübner et al., 2019), maize (Hirsch et al., 2014), *Brassica rapa* (Lin et al., 2014), *Brassica napus* (Hurgobin et al., 2018; Song et al., 2020), *B. oleracea* (Golicz et al., 2016b), *Sesamum indicum* (Yu et al., 2019), wheat (Montenegro et al., 2017), *Brachypodium distachyon* (Gordon et al., 2017), and *Cajanus cajan* (Zhao et al., 2020) have been mapped to open new horizons for the crop improvement studies. A detailed summary of the currently reported pan-genomes is summarized in Table 1.

**Table 1:** Summary of major crop pan-genomic studies.

**Crop**	**Year**	**Accessions**	**Ploidy level**	**Genome size**	**Assembly method**	**Outcrossing**	**Total pan-genes**	**Core %**	**Accessory %**	**References**
*Glycine max*	2020	26	Tetraploid	1011.6 Mb	*de novo*	-	57,492	50.1	49.9	Liu et al., 2020b
*Cajanus cajan*	2020	89	Diploid	622 Mb	Iterative	20%	55,512	86.6	13.4	Zhao et al., 2020
*Brassica napus*	2020	8	Tetraploid	1,033 Mb	*de novo*	-	105,672	56	42	Song et al., 2020
*Helianthus annuus*	2019	493	Diploid	3.6 Gb	*de novo*	-	61,205	95	5	Hübner et al., 2019
*Solanum lycopersicum*	2019	725	Diploid	950 Mb	*de novo*	0–5%	40,369	74.2	35.8	Gao et al., 2019
*Sesamum indicum*	2019	5	Diploid	554 Mb	*de novo*	-	15,409	58.21	41.79	Yu et al., 2019
*Oryza sativa*	2018	3010	Diploid	430 Mb	Map-to-pan	1–2%	48,098	48.5–58.3	41.7–51.5	Wang et al., 2018
*Oryza sativa*/*Oryza rufipogon*	2018	66	Diploid	430 Mb	*de novo*	1–2%/ 10–56%	42,580	61.9	38.1	Zhao et al., 2018a
*Brassica napus*	2017	53	Tetraploid	1.1 Gb	Iterative	28–30%	94,013	62.26	37.74	Hurgobin et al., 2018
*Triticum aestivum*	2017	18	Hexaploid	17 Gb	Iterative	1%	140,500	57.70	42.30	Montenegro et al., 2017
*Brassica oleracea*	2016	10	Diploid	650 Mb	Iterative	30%	61,379	81.3	18.7	Golicz et al., 2016b
*Zea mays*	2014	503	Tetraploid	2.4 Gb	Pan-transcriptomics	95%	41,903	39.12	60.88	Hirsch et al., 2014
*Glycine soya*	2014	7	Tetraploid	1 Gb	de-novo	5%	59,080	48.60	51.40	Li et al., 2014

In recent years, several visualization platforms have been designed for crop pan-genomes analyses like GET_HOMO LOGUES (Contreras-Moreira et al., 2017), PanViz (Pedersen et al., 2017), SplitMem (Marcus et al., 2014), RPAN (Sun et al., 2017), Pantools (Anari et al., 2019), ppsPCP (Tahir Ul Qamar et al., 2019), seq-seq-pan, ITEP (Jandrasits et al., 2018), EUPAN (Hu et al., 2017), PGAP-X (Zhao et al., 2018b), PanGP (Zhao et al., 2014), Micropan (Snipen and Liland, 2015), and PGAP (Zhao et al., 2012). These visualization tools permit analyzing the pan-genomes for retrieving the genes in databank, enabling access to PAV, gene annotation, gene expression, and genomic sequence information (Danilevicz et al., 2020). The design of advanced visualization tools is needed to allow a robust integrated examination of pan-genomes for future applications in crop improvement. A more detailed description of these analytical platforms is summarized in Table 2.

**Table 2:** List of some important tools for pan-genomic analysis.

**Tool**	**Year**	**Characteristics and functions**	**Web link**	**Platform**	**References**
Pantools	2019	A versatile tool for mapping the metagenomic and genomic reads in both prokaryotes and eukaryotes.	https://git.wur.nl/bioinformatics/pantools	Window, Linux	Anari et al., 2019
ppsPCP	2019	Detect presence/absence variations (PAV) and assembled comprehensive pan-genome	http://cbi.hzau.edu.cn/ppsPCP/	Linux	Tahir Ul Qamar et al., 2019
PGAP-X	2018	Analyze pan-genome profile curve, gene distribution analysis, genomic region variations, and comparative analysis of genome structure.	http://pgapx.ybzhao.com	Windows, Linux	Zhao et al., 2018b
EUPAN	2017	It can be applied to analyze the eukaryotic pan-genomes uses the R, C++, and Perl languages.	http://cgm.sjtu.edu.cn/eupan/index.html	Linux	Hu et al., 2017
PanViz	2017	Robust pan-genome analysis and visualization of variations in different genomic regions.	https://github.com/thomasp85/PanViz	Linux	Pedersen et al., 2017
GET_HOMOLOGUES-EST	2017	An R package software to categorized core and dispensable sequences and construct pan-genome matrices.	https://github.com/eead-csic-compbio/get_homologues/releases	Linux	Contreras-Moreira et al., 2017
RPAN	2017	Rich source for rice genomic research and breeding.	http://cgm.sjtu.edu.cn/3kricedb/	Linux	Sun et al., 2017
Micropan	2015	External source free computational pipeline and use R package for inclusive pan-genome analysis.	https://cran.r-project.org/web/packages/micropan/index.html	Windows, Linux	Snipen and Liland, 2015
PanGP	2014	Pan-genome profiling analysis, develop core genome, handle huge data set and user friendly.	http://PanGP.big.ac.cn	Windows, Linux	Zhao et al., 2014
SplitMem	2014	Graphical algorithm online web tool, which produced de Bruijn graph for pan-genome visualization.	http://splitmem.sourceforge.net	Linux	Marcus et al., 2014
PGAP	2012	It can be used to perform pan-genome profiling, gene cluster analysis, species evolution analysis, gene enrichment, and genetic variation analysis.	http://pgap.sf.net	Linux	Zhao et al., 2012

### Pan-Genomes and Crop Improvement

The crop pan-genomes studies enable us to catch up with the genes that are lost in reference genomes during crop domestication. Availability of the crop pan-genome that comprises all its CWRs, landraces, and cultivated varieties gives a well-defined system to gather all the information about genotypic and phenotypic variations and permit the identification of missing genes within the reference genomes (Danilevicz et al., 2020). Superior knowledge about accessory genome would help to screen the elite cultivars for abiotic and biotic stresses harboring stress-responsive genes (Bayer et al., 2019). Pan-genomes offer a great opportunity to understand the role of GD in genomic-based crop improvement. Fully annotated hexaploid wheat pangenome of 18 elite lines showed 140,500 ± 102 genes and 36 million intervariatel SNPs. The functional analysis of dispensable genes revealed their association with important agronomic traits (Montenegro et al., 2017). Similarly, several SNPs controlling the nine agronomic traits and variable genes for disease resistance have been identified in the pigeon pea pangenome that can be very useful for crop improvement (Zhao et al., 2020).

For example, SVs of 25 maize inbred lines have been studied and found a similar order among heterotic set and PAVs showing that SVs can be crucial for the identification of parentage for hybrid development (Darracq et al., 2018). The yield-related traits have been subjected to a rigorous selection during breeding, and the desired alleles for improved productivity have been identified in major crops like soybean (Concibido et al., 2003), rice (Thalapati et al., 2012), and wheat (Huang et al., 2003). Díaz et al. (2012) studied the genes controlling the flowering time in wheat and discovered that the genes show CNV. Similarly, FLC gene family regulates flowering in *B. oleracea* having variations in the CNV and found four core genes and two variable genes (Golicz et al., 2016b). The *Sub1A gene* regulating the submergence tolerance has been identified in rice under submergence (Schatz et al., 2014).

**Figure 4:**
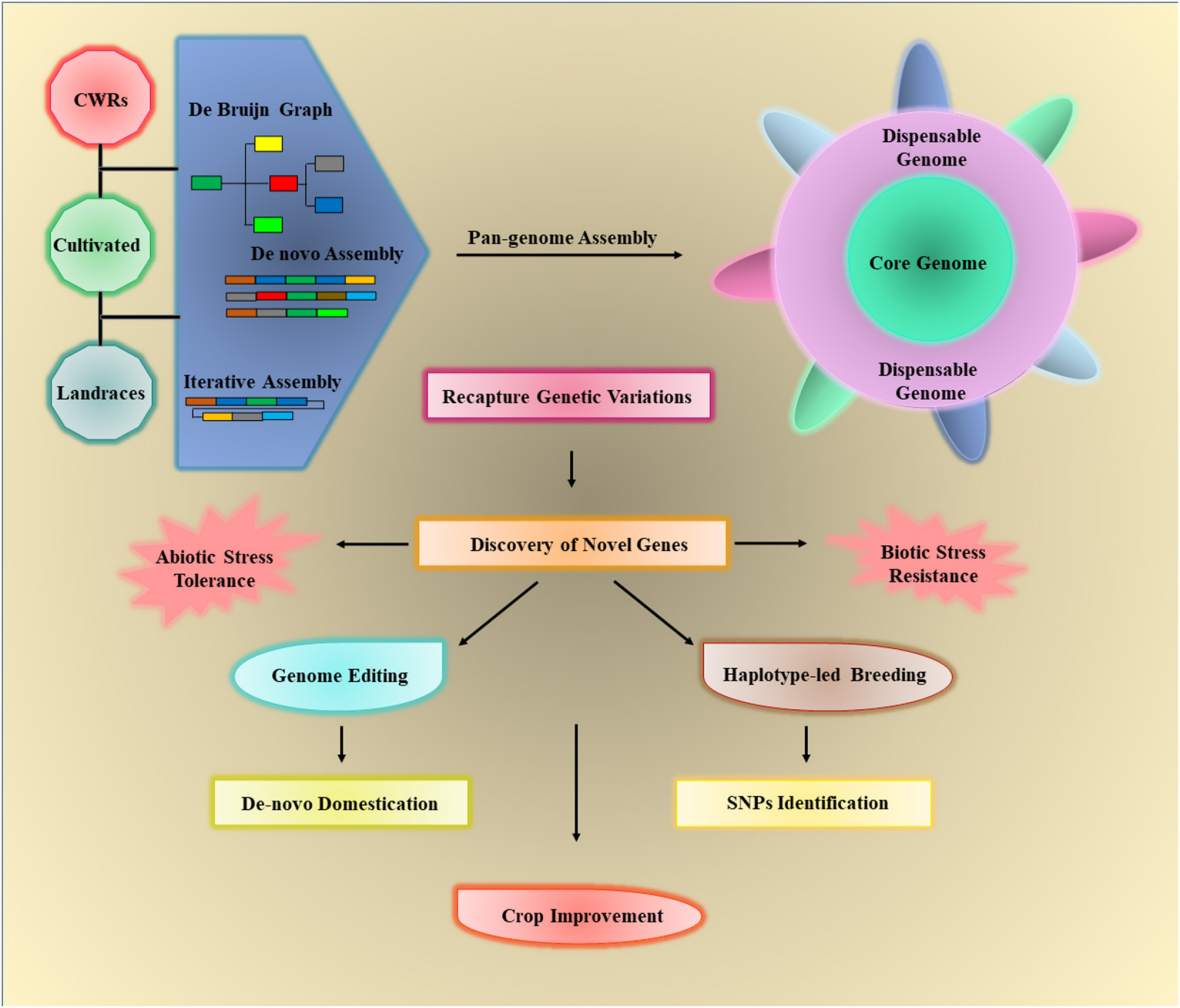
The crop wild relatives (CWRs), landraces, and cultivated varieties of crops can be used to assemble the crop pan-genomes *via* three approaches such as *de novo* assembly, de Bruijn graph, and iterative assembly. The core genome includes all the genes of individuals while the dispensable or assessor genome contains all remaining genes, which are not necessary to present in all individuals. Pan-genomes can be used to identify different structural variations (SVs) in any individual and detect novel genes that are lost in cultivated varieties during the breeding process. The elucidation of desired traits/genes can be used for crop improvement by providing biotic/abiotic stress tolerance through haplotype-based breeding and *de novo* domestication.

Several biotic stress-responsive genes have revealed the PAVs in a large number of species (Cook et al., 2012). Recently, Dolatabadian et al. (2020) analyzed the pan-genome of 50 *B. napus* genotypes to characterize the disease-resistant genes. The pan-genome analysis unveiled 1,749 resistance genes, from which 753 are dispensable and 996 are core gene while 368 genes are not detected in the reference genome. Furthermore, SNPs studies revealed 15,318 unique hotspots within 1,030 resistance gene orthologs and identified 106 putative QTLs related to blackleg resistance (Dolatabadian et al., 2020). Sunflower pangenome identified 61,205 genes across a diverse range of wild and cultivated species. Functional annotation of biotic stress-related genes showed an allelic variation for disease resistance including downy mildew (Hübner et al., 2019). The resistance gene orthologs vary among different cultivars, and capture 59 candidate genes that are linked with the Fusarium wilt, clubroot, and Sclerotinia-resistant QTLs in *B. oleracea* pan-genome (Bayer et al., 2019). The progressive information gathered from these studies can be used to develop improved crop cultivars. The workflow of pan-genome assembly and its exploitation for crop improvement is illustrated in Figure 4.

### A Breakthrough Is Still Needed

Numerous pitfalls remain to be dealt with in achieving economical, robust, and perfectly annotated pan-genome assembly. First, the main prerequisite for pan-genomic analysis is the accessibility to a well-annotated reference genome. However, the major drawback in transferring the adjoining sequences is the short genomic reads that cause hindrance in repetitive region assembly. Novel techniques like single-molecule sequencing can offer longer reads but have low accuracy. With the advent of highly sophisticated algorithms tools, the assembly of long reads has become easy and robust, which result in the assembly of high-quality genomes. The incorporation of long reads can help in resolving the highly complex polypoid genomes and repetitive motifs that are quite challenging to read short sequences, and this strategy can be useful for the assembly of pan-genomes. Another crucial aspect for the pan-genome analysis is the useful association among phenotypes and functional data sets. Only accurate functional data will create a linkage map between trait diversity and pan-genome.

Second, the size and complexity of several genomes pose a big hurdle for developing the visualization tools for pan-genomic interpretations. To tackle this, scientists have come up with cloud-based solutions to assist the availability of pan-genome visualization tools. The cloud-based system could harness the multi-omics approaches and established a huge platform for data sharing, pan-genomic analysis, and developing the standardized protocols for the pan-genome studies. Third, the presentation and storage of large data sets of pan-genome results are also challenging tasks. Databases can store a large number of pan-genomic data, including transposable elements, noncoding RNAs, indels, and SNPs. Developing a coordinate system like SuperGenome requires to be upgraded to address the challenges related to presentation and storage. Fourth, the information collected from the pan-genome needs GWAS/QTL analysis and re-sequencing to unravel the candidate genes for crop improvement. Thus, overcoming these hurdles to develop inclusive genomic resources would beneficial to expand the pan-genomic assemblies to other crop species. Furthermore, the applications of ML and deep learning (DL) in pan-genomic research may facilitate to cross the barriers of pan-genome construction and visualization as ML tools can autonomously detect the sequence reads in huge data sets. Also, a complete information flow reflecting gene-transcriptome-metabolome-epigenome-phenome can provide an excellent platform to study a phenotypic variation within different microenvironments. As the omics tools continue to attain more precision and sensitivity for analytical investigation, the era of crop “pan-omics” is not far away. The upcoming future is expecting to see the data science explosion, where “Crop Pan-omes” studies will be one of the key players in giving a new direction to future agriculture.

## The Era of Plant Genome Editing

To date, classical breeding is speedier in comparison to 50 years ago but still inadequate to accelerate the agricultural production with respect to the global demands (Breseghello and Coelho, 2013; Voss-Fels et al., 2019). In addition, mutation breeding and transgenic technology can be applied to introduce novel genes for crop improvement, but GMOs are banned in many countries because of public health safety and regulatory concerns. It normally takes 10–12 years to design a crop cultivar through conventional, mutational, and transgenic breeding (Razzaq et al., 2019b).

The rise of first-generation genome engineering nulceases, like transcriptional activator-like effector nucleases (TALENs) and zinc finger nucleases (ZFNs), brought a revolution in plant research and accelerated the plant research (Lloyd et al., 2005; Cermak et al., 2011). Genome editing has been applied to generate insertion/deletion (indels), substitution, replacement, and overcome all the concerns of nonspecific, cross species, and unstable integration of foreign DNA into the host cell (Kim and Kim, 2014). Although, ZFNs and TALENs have been extensively applied for site-specific plant genome editing in the past but certain drawbacks, like cumbersome cloning and vector construction protocols, large size, ineffective delivery methods, repetitive nature, less specificity, and large off-target, limit their use today (Puchta, 2017).

### Broadening the CRISPR/Cas Toolbox

Clustered regularly interspaced short palindromic repeat/CRISPR-associated system is the most fascinating and ground-breaking technology for genome editing (Jinek et al., 2012). Continuous efforts are in progress to minimize the drawbacks of the CRISPR/Cas systems in plants and to develop next-generation genome editing tools.

The present classification of a CRISPR toolkit is incomplete as new classes of variants are discovered continuously (Koonin et al., 2017). A CRISPR/Cas system is still evolving and has yet to fulfill its potential. Currently, there are two major classes (classes 1 and 2), six types, and more than 30 subtypes of the CRISPR system according to their respective signature protein. The class 1 includes multiple Cas effector proteins to perform many tasks and comprises type I, III, and IV with the corresponding proteins like Cas3, Cas10, and Csf1 (Makarova et al., 2015). Whereas the class 2 system with only single signature protein is the most extensively adopted genome editing system and consists of type II (Cas9), V [Cas12a (Cpf1), Cas12b, Cas12c, Cas12d (CasY), Cas12e (CasX), Cas12g, Cas12h, Cas12i, Cas14a, Cas14b, Cas14c], and VI [Cas13a (C2c2), Cas13b (C2c6), Cas13c-d] systems (Koonin et al., 2017). Several Cas orthologs as depicted in Table 3 have been discovered to overcome the bottlenecks in the CRISPR/Cas system.

**Table 3:** List of different Cas orthologs used for plant genome editing.

**Cas type**	**Organism**	**PAM**	**Size**	**Cutting site**	**gRNA**	**Target**	**Plant species**	**Characteristics**	**References**
SpCas9	*Streptococcus pyrogenes*	NGG	1,368 bp	5′-PAM	20 bp	dsDNA	Several plants	Need long crRNA+tracrRNA	Jinek et al., 2012
SpCas9 QQR1	*Streptococcus pyrogenes*	NAAG	1,372 bp	5′-PAM	20 bp	dsDNA	-	Altered PAM sequence	Cong et al., 2013
SpCas9 VRER	*Streptococcus pyrogenes*	NGCG	1,372 bp	5′-PAM	20 bp	dsDNA	Rice	Altered PAM sequence	Kleinstiver et al., 2015
SpCas9-NG	*Streptococcus pyrogenes*	NG	1,372 bp	5′-PAM	-	DNA	Rice	Altered PAM sequence, greater ability of base editing and gene regulation	Ren et al., 2019
SaCas9	*Staphylococcus aureus*	NNAGRRT	1,053 bp	5′-PAM	21 bp	DNA	Rice and citrus	Reduce off-targets and excellent *in vivo* genome editing	Kaya et al., 2016
FnCas9	*Francisella novicida*	NGG	1,629 bp	5′-PAM	20 bp	DNA	-	Reduce off-targets	Hirano et al., 2016
ScCas9	*Streptococcus canis*	NNG	1,379	5′-PAM	20 bp	DNA	-	Altered PAM sequence and reduce off-targets	Chatterjee et al., 2018
Nme Cas9	*Neisseria meningitidis*	NNNNGATT	1,082	5′-PAM	24 bp	DNA	-	Reduce off-targets and need longer PAM	Lee et al., 2016
BlatCas9	*Brevibacillus laterosporus*	NNNNCND	1,092	5′-PAM	20 bp	DNA	Maize	Enhance specificity	Karvelis et al., 2015
St1Cas9	*Streptococcus thermophilus*	NNAGAAW	1,121	5′-PAM	20 bp	DNA	*Arabidopsis*	Reduce off-targets	Steinert et al., 2015
St3Cas9	*Streptococcus thermophilus*	NGGNG	1,409	5′-PAM	20 bp	DNA	-	Multiple domains and induce dsDNA breaks	Cong et al., 2013
HypaCas9	*Streptococcus pyogenes*	NGG	1,368	5′-PAM	20 bp	DNA	Rice	Increased specificity	Chen et al., 2017
eHypa-Cas9	*Streptococcus pyogenes*	NGG	1,368	5′-PAM	20 bp	DNA	Rice	Increased specificity	Liang et al., 2018
CjCas9	*Campylobacter jejuni*	NNNNRYAC or NNNNACAC	984	5′-PAM	22 bp	DNA	-	Greater mutation frequency	Kim et al., 2017
xCas9 3.7	*Streptococcus pyogenes*	GAT, GAA, NG	1,368	5′-PAM	-	DNA	Rice	Altered PAM and increased specificity	Zhong et al., 2019
CasX	*Planctomycetes and Phyla Deltaproteobacteria*	TTCN	980	5′-PAM	23 bp	DNA	-	Increased specificity	Burstein et al., 2017
AsCpf1	*Acidaminococcus sp*.	TTTN	1,307	3′-PAM	24 bp	DNA	-	Increase editing efficiency	Yamano et al., 2016
Cpf1	*Francisella1 and Prevoltella*	TTTV	-	5′-PAM	20 bp	DNA	Rice and *Arabidopsis*	Need long sgRNA and lacks HNH domain	Endo et al., 2016
FnCpf1	*Francisella novicida*	TTTV and TTV	-	5′-PAM	20 bp	DNA	Rice	Enhanced efficiency and altered PAM	Zhong et al., 2018
Cas12a	*Acidaminococcus sp*.	TTTV	1,307	5′-PAM	20 bp	DNA	-	Altered PAM	Jeon et al., 2018
LbCas12a RR	*Francisella1 and Prevoltella*	CCCC and TYCV	1,228	5′-PAM	20 bp	DNA	Rice	Altered PAM	Kleinstiver et al., 2019
AsCas12a RVR	*Francisella1 and Prevoltella*	TATV	1,307	5′-PAM	20 bp	DNA	-	Altered PAM	Kleinstiver et al., 2019
FnCas12a RVR	*Francisella1 and Prevoltella*	TWTV	1,300	5′-PAM	20 bp	DNA	Rice	Altered PAM	Zhong et al., 2018
MbCas12a RR	*Francisella1 and Prevoltella*	TCTV and TYCV	1,373	5′-PAM	20 bp	DNA	-	Altered PAM	Tóth et al., 2018
Cas13 (C2c2)	*Leptotrichia shaii*	Not needed	1,440	-	28 bp	ssRNA	-	Cleaved RNA	Abudayyeh et al., 2016
AacC2c1	*Alicyclobacillus acidoterrestris*	T-rich PAM	1,227	5′-PAM	20 bp	DNA	-	Bi-lobed endonucleases	Liu et al., 2017
Cas14	Archaea	-	400–700	-	-	ssDNA	-	Restrictive sequence not required for ssDNA cleavage	Harrington et al., 2018

Naturally, Cas9 is found in Streptococcus pyogenes (SpCas9) and contains three subunits: a Cas9 protein, CRISPR RNA (crRNA), and trans-activating crRNA (tracrRNA) (Jinek et al., 2012; Mali et al., 2013). Cas9 comprises two lobe-like structures: a recognition domain (REC) linked with a nuclear domain (NUC). The NUC lobe consists of two catalytic sites like HNH and RuvC that target the protospacer adjacent motif (PAM) present at 3 bp upstream of the desired DNA region. The mechanism of CRISPR/Cas9 editing initiates by designing 20-bp guide RNA (gRNA) to form gRNA/Cas9 assembly and recognize PAM site to produce double-standard breaks (DSBs) at a specific site (Cong et al., 2013). CRISPR/Cas12a is considered as another important Cas ortholog, which is also called as Cpf1. It is an RNA-based editing tool and presents some inimitable characters in contrast to a CRISPR/Cas9 system. Cpf1 needs a T-rich spacer region at 5'-end having 42-nt crRNA and create DSBs with staggered ends (Zetsche et al., 2015). For example, Cpf1-based genome editing was performed in allotetraploid cotton, and the results indicated zero off-target cleavage with 87% editing efficiency (Li et al., 2019a). CRISPR/Cas9 and CRISPR/Lsh Cas13a have been applied against RNA potyvirus to make disease-resistant plants and can be applied against many other invading viruses (Aman et al., 2018). CRISPR/Cas14a is a highly ideal tool to target the plant single-standard DNA viruses like Nanoviridae and Geminiviridae families. It has the ability to develop viral-resistant crops by allowing unrestricted cleavage without any dependency on the sequence (Khan et al., 2019).

### Engineering Crops for Improved Stress Resilience

A tremendous progress in plant genome engineering has been achieved by exploiting the CRISPR/Cas system for crop improvement (Li et al., 2013; Shan et al., 2013). CRISPR/Cas technology is revolutionizing the plant breeding due to its immense application to develop climate-resilient crops (Puchta, 2017). There are numerous studies reported for improved agronomic traits and stress tolerance to abiotic/biotic stresses (Table 4). For example, drought is the most damageable abiotic stress causing severe loss to crop production. A CRISPR/Cas9 system was used to produce the knockout mutants of *SlLBD40* in tomato subjected to drought stress. The mutant lines showed improved water-holding capacity as compared to the *SlLBD40* overexpressing line. The results demonstrated that the *SlLBD40* gene negatively regulates drought stress in tomato (Liu et al., 2020a). CRISPR/Cas9 was employed to study the effect of *SlNPR1* mutants under drought stress and revealed that the knockout tomato lines were highly susceptible to drought stress (Li et al., 2019b). Ogata et al. (2020) applied CRISPR/Cas9-mediated frame shift mutations to develop the rice mutant lines for the *OsERA1* gene under drought stress. The results showed an increase in tolerance to drought and positively induce primary root development under normal conditions. Ramírez Gonzales et al. (2021) provided the evidence about the *StCDF1–StFLORE* locus that regulates the water homeostasis and vegetative growth in potato by disrupting the *StFLORE* using a CRISPR/Cas9 system. Loss of function of this gene resulted in enhanced drought tolerance. Pan et al. (2020) reported that the gene *ZmSRL5* is very vital for a cuticular wax structure, which protects the maize plant from different stresses. Loss of functional mutant progenies of maize showed that the *ZmSRL5* gene involved in drought response by keeping the structure of cuticle wax intact.

**Table 4:** Applications of clustered regularly interspaced short palindromic repeat/CRISPR-associated (CRISPR/Cas) system to engineered abiotic/biotic stress tolerance.

**Crop**	**Gene**	**Stress**	**Target result**	**Vector delivery**	**References**
**Abiotic stress**
Tomato	*SlLBD40*	Drought	Knockout	*A. tumefaciens*	Liu et al., 2020a
Rice	*OsERA1*	Drought	Knockout	*Agrobacterium tumefaciens*	Ogata et al., 2020
Tomato	*SlNPR1*	Drought	Knockout	*Agrobacterium tumefaciens*	Li et al., 2019b
Potato	*StFLORE*	Drought	Knockout	*Agrobacterium tumefaciens*	Ramírez Gonzales et al., 2021
Maize	*ZmSRL5*	Drought	Knockout	*Agrobacterium tumefaciens*	Pan et al., 2020
Rice	*OsRR22*	Salinity	Knockout	*Agrobacterium tumefaciens*	Zhang et al., 2019
Tomato	HyPRP1 domain	Salinity	Knockout	*Agrobacterium tumefaciens*	Tran et al., 2020
Rice	*HDA710*	Salinity	Knockout	*Agrobacterium tumefaciens*	Ullah et al., 2020
Rice	*OsNAC041*	Salinity	Knockout	*Agrobacterium tumefaciens*	Bo et al., 2019
Soybean	*GmNAC06*	Salinity	Knockout	*A. rhizogenes*	Li et al., 2021
Rice	*OsNAC006*	Multiple	Knockout	*Agrobacterium tumefaciens*	Wang et al., 2020a
Rice	*OsDST*	Multiple	Knockout	*Agrobacterium tumefaciens*	Santosh Kumar et al., 2020
**Biotic stress**
Rice	*Xa13*	Bacterial blight	Knockout	*Agrobacterium tumefaciens*	Li et al., 2020a
Rice	*AvrXa7*	Bacterial blight	Knockout	*Agrobacterium tumefaciens*	Zafar et al., 2020
Rice	*Os8N3*	*Xanthomonas oryzae*	Knockout	*Agrobacterium tumefaciens*	Kim et al., 2019b
Rice	*OsSWEET14*	*Xanthomonas oryzae*	Knockout	*Agrobacterium tumefaciens*	Zeng et al., 2020
Tomato	*SlJAZ2*	Bacterial speck	Knockout	*Agrobacterium tumefaciens*	Ortigosa et al., 2019
Cassava	*AC2, AC3*	African cassava mosaic virus	Interference	*Agrobacterium tumefaciens*	Mehta et al., 2019
Cassava	*nCBP-1* and *nCBP-2*	Cassava brown streak virus	Knockout	*Agrobacterium tumefaciens*	Gomez et al., 2019
Soybean	*GmF3H1, GmF3H2* and *GmFNSII-1*	Soybean mosaic virus	Knockout	*Agrobacterium tumefaciens*	Zhang et al., 2020
Tomato	*PMR4*	Powdery mildew	Knockout	*Agrobacterium tumefaciens*	Martínez et al., 2020

Salinity tolerance in important crop species can also be attained by exploiting CRISPR/Cas9-mediated genome editing. CRISPR/Cas9 technology was executed to generate *OsRR22* mutant lines of rice, which exhibit enhanced salt tolerance at the seedling stage (Zhang et al., 2019). Regulatory domain editing of multidomain proteins is another important aspect that can be used to engineer the negatively regulated domains associated with abiotic stresses. Tomato hybrid proline-rich protein 1 (*HyPRP1*) domain was targeted successfully by using multiplex genome editing *via* CRISPR/Cas9, which is a negative regulator for salinity stress. The results revealed that the elimination of this domain produces tomato with high salinity tolerance (Tran et al., 2020). Ullah et al. (2020) disrupted the *HDA710* gene through CRISPR/Cas9, and the mutant lines displayed reduced abscisic acid sensitivity and increased salinity tolerance in rice. A large number of transcription factors are involved in the salinity tolerance mechanism including NAC transcription factors. A CRISPR/Cas9 system was used to develop the knockout mutants of the *OsNAC041* gene in rice. The study indicated a direct function of the *OsNAC041* gene under salt stress and can offer an excellent potential to target other NAC genes for rice-resistant breeding (Bo et al., 2019). Similarly, another NAC gene *GmNAC06* was targeted by using CRISPR/Cas9-based gene editing and overexpression technology in soybean, and the results demonstrated that the *GmNAC06* gene improved the salinity tolerance by alleviating ROS effects, accumulating glycine betaine and proline, and maintaining ionic homeostasis (Li et al., 2021).

Wang et al. (2020a) executed CRISPR/Cas9-mediated genome editing to mutate the *OsNAC006* gene in rice and the mutant lines showing a high susceptibility to heat and drought stress. Likewise, CRISPR/Cas9 was applied to develop the mutant alleles for the *OsDST* gene, and the result exhibited a reduction in stomatal density while increasing tolerance against drought, salinity, and osmotic stress without damaging the rice grain yield (Santosh Kumar et al., 2020).

Clustered regularly interspaced short palindromic repeat/CRISPR-associated gene editing also provide a great opportunity to combat against plant pathogens and invading organisms efficiently. Such a resistance to bacterial blight disease was improved in rice by editing the promoter region of *Xa13* gene. The knockdown progenies were transgene-free edited plants and showed an increase in resistance to bacterial blight (Li et al., 2020a). In a similar report, the *AvrXa7* gene was the target for bacterial blight resistance in basmati rice (Zafar et al., 2020). A CRISPR/Cas9 system was developed by two independent groups to manipulate the *Os8N3* and *OsSWEET14* gene in rice. The results showed that the mutations were transferred in successive progenies, and homozygous knockouts demonstrated an increased resistance to *Xanthomonas oryzae* pv. *oryzae* without any yield in plenty (Kim et al., 2019b; Zeng et al., 2020). Ortigosa et al. (2019) designed a CRISPR/Cas9 system to develop bacterial speck-resistant tomatoes. In this experiment, *SlJAZ2* functional ortholog, which was found in stomata, was edited, which provide resistance to bacterial speck-causing agent *Pseudomonas syringae* pv.

Clustered regularly interspaced short palindromic repeat/CRISPR-associated genome editing can also be applied to cope with the different plant viruses. Mehta et al. (2019) developed a CRISPR/Cas9 interference system to target *AC2* and *AC3* genes and to have an engineered resistance against African cassava mosaic virus. This system is very useful to cope with the Geminiviruses attack in plants. The resistance against soyabean mosaic virus has been achieved by targeting the genes involved in metabolic pathways of isoflavone, including *GmF3H1, GmF3H2*, and *GmFNSII-1* using the multiplex CRISPR/Cas9 system (Zhang et al., 2020).

Martínez et al. (2020) examined the role of the tomato *PMR4* gene against powdery mildew by mutating it *via* a CRISPR/Cas9 tool. The mutant lines exhibited a higher susceptibility to fungal infection as compared to the normal plants. This evidence can be useful to characterize and analyze S-genes under different fungal pathogens. CRISPR/Cas9 was used to produce the knockouts for *nCBP-1* and *nCBP-2* genes to study the resistant mechanism against cassava brown streak virus. The knockouts lines displayed little symptoms and reduced disease severity in contrast to control lines (Gomez et al., 2019).

### Recent Innovations in CRISPR/Cas System

**Figure 5:**
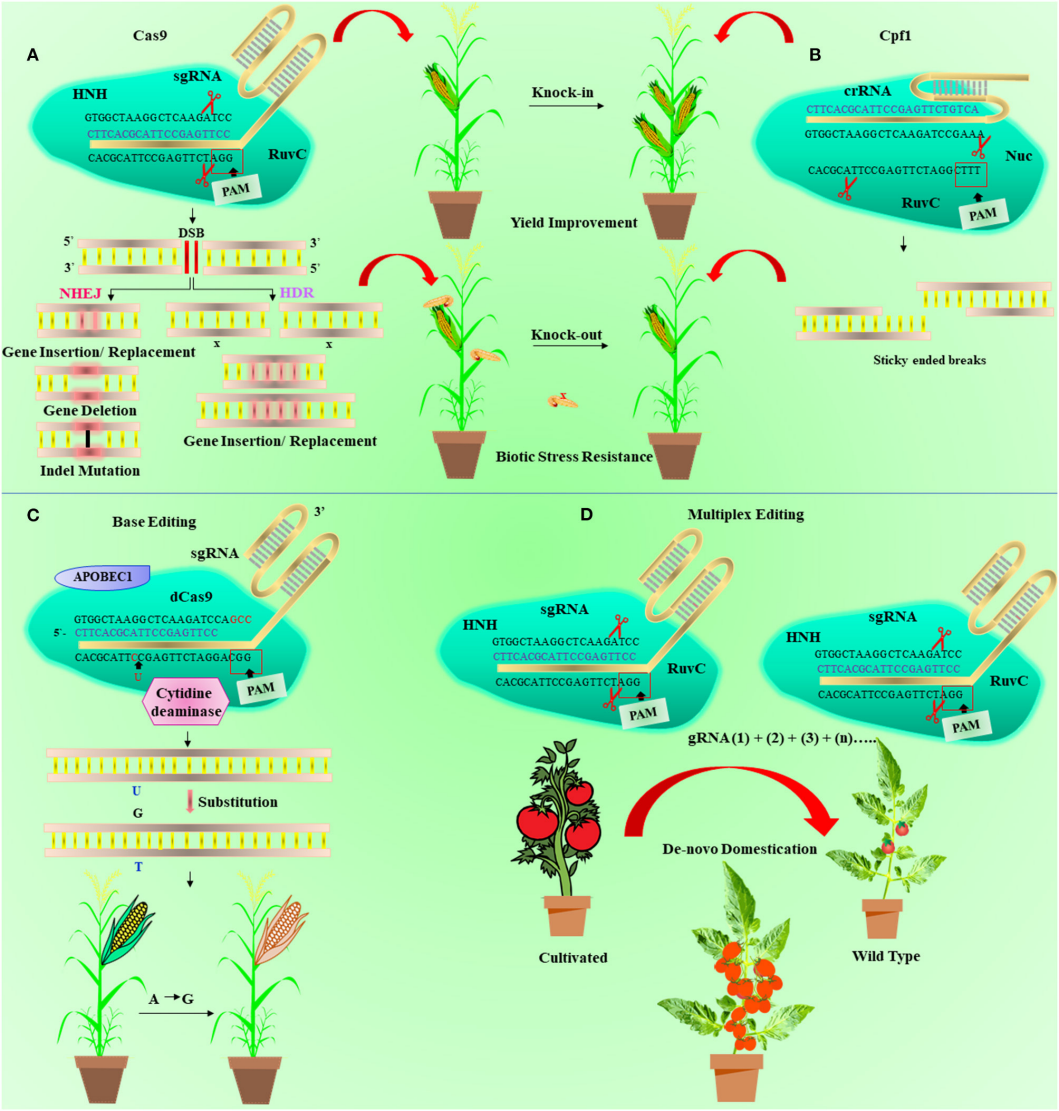
Diagrammatic illustration of the base editing, clustered regularly interspaced short palindromic repeat/CRISPR-associated 9 (CRISPR/Cas9) and Cpf1 mechanism, and *de novo* domestication. **(A)** In the CRISPR/Cas9 mechanism, Cas9 protein is guided and activated with the help of CRISPR RNA (crRNA) and trans-activating CRISPR RNA (tracrRNA), respectively, to produce double-standard breaks (DSBs) in DNA. The single-guide RNA (sgRNA) (blue) is developed with the grouping of tracrRNA and crRNA and identifies the 20-nucleotide (orange) target sequence. This makes a complex of Cas9-sgRNA, which moves along the target site and cuts double-standard DNA 3 bases upstream of protospacer adjacent motif (PAM) through HNH and RuvC domains. The DSBs are reconstructed *via* nonhomologous end-joining (NHEJ) or homology-directed repair (HDR) pathway. **(B)** Shows the Cpf1 mechanism that recognize the 24-nucleotide target sequence (blue) of crRNA and cleaves five nucleotides opposite to T-rich (TTTN) spacer at 5′ end. **(C)** Representing the base editing in which dead Cas9 (dCas9) is associated with cytidine deaminase (brown). It is directed by sgRNA (blue) for base substitute at target sequence (orange) distal to PAM site at 3' end. **(D)** depicted the *de novo* domestication process in wild plant using multiplex genome editing. Multiple guide RNA (gRNA) can be used to edit more than one gene simutanelously linked to some agronomic traits.

Without any doubt, the next-generation CRISPR/Cas technology continuously opens up new avenues in plant breeding research. Some breakthrough strategies were reported for the CRISPR/Cas system that removes certain constraints prevailing in genome editing as shown in Figure 5. Recent advancement in the CRISPR/Cas toolbox results in the improvement of several novel features, like target specificity, broader target range, minimizing off-targets, precise nuclease activity, various PAM sites, and efficient delivery methods (Koonin et al., 2017). It is the preferred editing system for carrying out other genetic modifications such as probing mutation patterns (Jia et al., 2018), the introduction of exogenous genes (Collonnier et al., 2017), gene regulation (Qi et al., 2013), and cell imaging (Xue and Acar, 2018).

For example, Maher et al. (2020) reported a protocol to remove all the barriers caused by the laborious and time-consuming protocols of tissue culture. They have developed *de novo* meristem induction by transferring all the editing machinery into the somatic cells to produce the shoots having targeted manipulations (Maher et al., 2020). Ren et al. (2019) developed a didirectional promoter (BiP) system to express gRNA and Cas9 cassettes in the opposite direction to enhance the editing effectiveness of about 75.9–93.3% in rice. Decaestecker et al. (2019) constructed a CRISPR-TSKO system to produce tissue-specific knockout mutants to overcome the pleiotropic effect of a mutated gene. This will open up new possibilities for crop improvement to target a tissue-specific gene (Decaestecker et al., 2019). The editing efficiency of Cas nucleases is greatly influenced by the preferred PAM sites as it has a limited range to target the GC-rich region. Recently, Ren et al. (2021) designed a PAM-free editing tool by developing the CRISPR-SpRY toolbox in rice. It can resolve the PAM sites' limitations to target a wide range of specific sequences in the DNA molecule. Uranga et al. (2021) engineered potato virus X to construct a vector delivery system for multiple gRNAs and achieved 80% indels mutation rate in targeting the *Nicotiana benthamiana* genes. Also, the virus-free lines can be screened from the progeny developed *via* infected seeds or tissues that demonstrate greater biallelic mutations.

#### Base Editing

Production of the precise point mutations in plant genomic base editing is an emerging strategy to disrupt a single base using CRISPR/nCas9 (Cas9 nickase) attached with cytidine deaminase. An efficient system called base editor 3 (BE3) has been developed for cotton to create the targeted base substitutions with the mutation rate of 26.67–57.78% (Qin et al., 2020). A novel adenine base editor (ABE) was designed by Li et al. (2018a) to produce herbicide-resistant wheat and rice plants. ABE was enabled G to C and A to T point mutations with 59.1% successful rate in regenerated lines (Li et al., 2018a). Sretenovic et al. (2020) engineered the iSpyMacCas9 tool for the targeted mutation at A-rich PAM sites that substitute A to G and C to A base. The constructed vector system is well-suited for gateway cloning and also very compatible for all types of gRNA. This platform can be used to improve other editing systems like CRISRP activation, CRISPR interference, homology-directed repair (HDR) system, and prime editing. Plant virus can also be used for a fast and an efficient delivery of CRISPR/Cas machinery for virus-induced editing. While the APOBEC3-Cas9 fusion-induced deletion was designed to cleave 5′-deaminated C bases in rice and wheat protoplasts (Wang et al., 2020b).

#### Prime Editing

Recently, prime editing is emerged as an ideal toolbox that can remove all the previous hurdles and produces insertion, deletions, and base substitutions without generating DSBs (Marzec et al., 2020). Xu group developed the first prime-editing system in rice by designing the plant prime editors (pPE2) toolkit to induce mutations at different genomic regions with a frequency of 0–31.3% (Xu et al., 2020a). After this study, a huge wave of genome editing using prime editing have been conducted in different crops (Hua et al., 2020). For example, a prime editor system was optimized by promoter and codon to introduce insertion, deletions, and point mutations in wheat and rice protoplast (Lin et al., 2020), generate single and multiple base edits in rice (Xu et al., 2020b) having the mutation rate of 21.8 and 26%, respectively. In later experiment, Lin et al. (2021) designed two prime-editing gRNAs, which led to an increase in editing efficiencies from 2.9-fold to 17.4-fold in rice. Furthermore, the prime-editing system was executed to target both endogenous and exogenous genes to produce homozygous and heterozygous mutated lines with minimum off-targets and developed herbicide-resistant lines of rice through base substitutions (Butt et al., 2020; Li et al., 2020b).

#### *De novo* Domestication

*De novo* domestication is another important breeding pipeline benefited by the powerful technology of CRISPR/Cas. *De novo* domestication can be used to exploit the GD and has a great potential to introduce desriable traits in CWRs (Razzaq et al., 2021). *De novo* domestication of four wild cultivars of tomato was performed by mutating the four genes *SlWUS, SlCLV3, SP5G*, and *SP* through CRISPR/Cas9-mediated multiplex genome editing to develop improved tomato fruits (Li et al., 2018b). Similarly, Zsögön et al. (2018) executed *de novo* domestication of wild tomato by disrupting the six genes related to useful agronomic traits using multiplex editing, which controls the nutritional and yield-related traits. The engineered wild progenies showed 500% increase in the accumulation of lycopene compared to the cultivated species. Also, they exhibited a 3-fold greater fruit size and 10-fold extra fruit number as compared to their wild parents (Zsögön et al., 2018). Recently, Yu et al. (2021) established an efficient transformation system in allotetraploid rice. The *de novo* domestication strategy was used for improving the six agronomically important traits in allotetraploid rice. *De novo* domestication using next-generation genome editing tools can provide an alternative strategy to explore the GD of CWRs for crop improvement.

#### Beyond Editing

Apart from genome editing, the CRISPR/Cas toolbox can be applied to regulate gene expression and epigenome editing. A newly fine-tuning gene expression regulation system has created to control the translation process in plants. A CRISPR/Cas9 system with the modified protocol is used to edit the upstream open reading frames to produce transgene-free mutated plants. This approach can be utilized to study the functions of several genes and facilitate rapid crop improvement programs (Si et al., 2020). Another genome editing system named as Cas12b has been designed for site-specific genome manipulation in plants. It is considered as the third most exceptional CRISPR tool after Cas12a and Cas9 system. It also has an excellent potential for gene regulation through transcriptional activation and repression mechanism in plants (Ming et al., 2020). Nuclease-dead Cas9 (dCas9) allows programmable regulation of multiple genes *via* transcriptional effectors without damaging the target site. CRISPR/dCas9 has vast applications, such as DNA-free genome engineering, live-cell chromatin imaging, chromatin topology, epigenome editing, and gene regulation, in plants (Moradpour and Abdulah, 2020).

### Technological Barriers and Public Concerns for CRISPR Technology

The major drawback in the applications of CRISPR-mediated engineering for plant breeding is not experimental or technical but consumer acceptance, public concerns, and strict regulatory affairs for the approval of edited crops. Technological improvement in genome editing tools would develop the similar traits just like the traits produced during conventional breeding in nature. However, CRISPR/Cas systems could be applied to incorporate foreign genes into the host genome, but this can easily be captured. Therefore, genome-edited crops developed through the CRISPR/Cas system should not be regarded as GM crops. Also, the use of CRISPR/Cas technology is still under strict constraints due to the ban imposed by European countries and cumbersome regulatory protocols adopted by USA, Australia, and Canada to ensure the biosafety of genome-edited crops. All these regulatory barriers will overcome only with a strong political determination and consensus among all stakeholders of different countries to consider the CRISPR-edited plants as non-GMOs.

**Figure 6:**
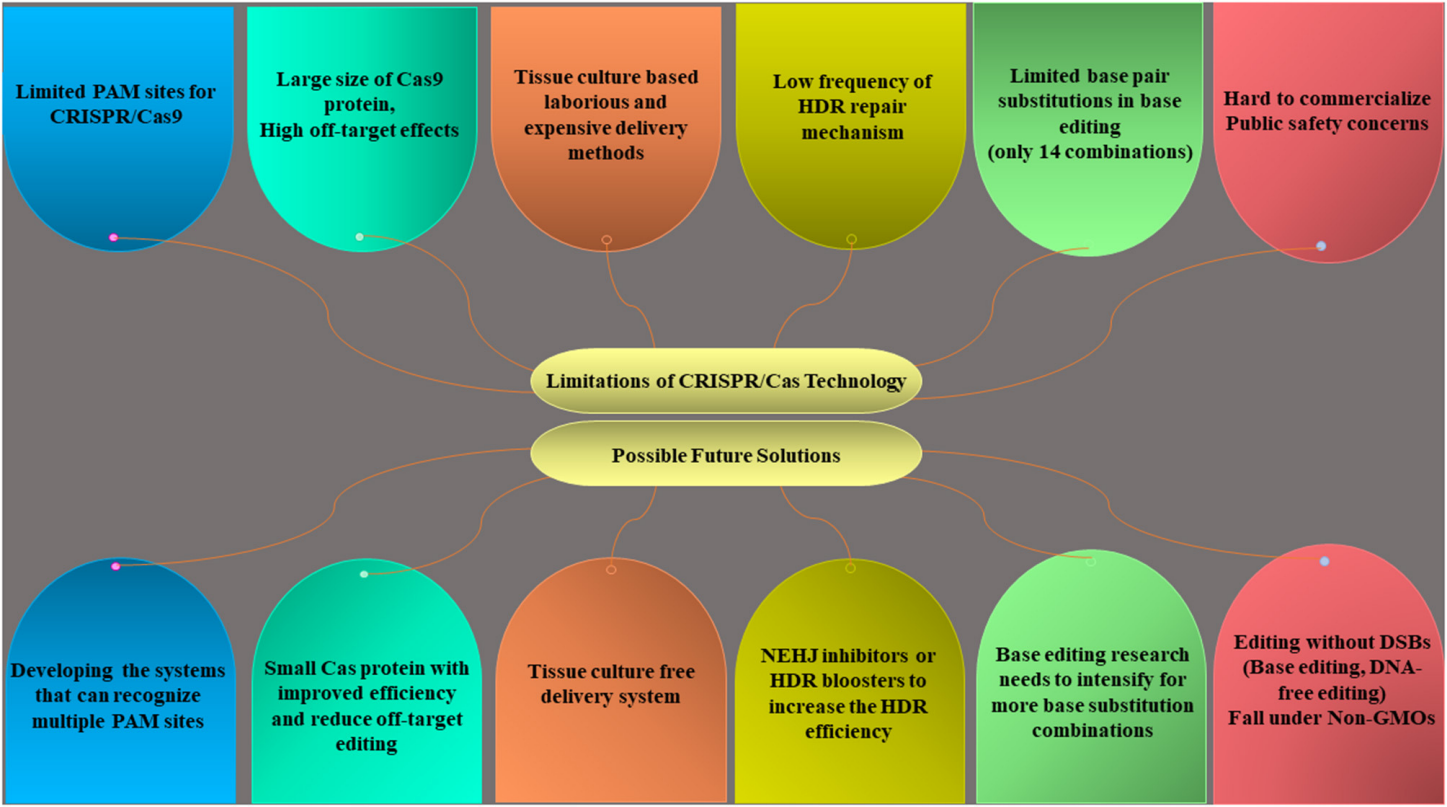
Limitations of the CRISPR/Cas system and a future way forward to develop ideal editing systems.

There are still remaining certain bottlenecks that need to be fixed to exploit the full potential of the CRISPR/Cas system (Figure 6). For example, SpCas9 has a larger size, greater off-target mutations, and can only detect the NGG PAM sequence that limits its use. The emergence of other Cas orthologs like Cpf1, Cas13a, and Cas14 (a,b) can solve this problem due to their small size, broader PAM sites, and reduced off-target effects. The establishment of tissue culture-free, transient CRISPR system like prime editing is required to make this technology in a more robust and simpler way. The use of nonhomologous end-joining (NHEJ) inhibitors or HDR boosters can improve the HDR efficiency but yet to be reported in plants. In future, the next-generation CRISPR systems can offer sustainable agriculture production by overcoming the technical and regulatory barriers.

## Next-Generation Plant Phenotyping Platforms

Plant trait phenotyping has been vital for successful crop domestication since thousands of years. The word “phenome” implies the whole phenotypic profile of any plant, and a phenotype is the blend of evident genomic expression with respect to its environment (Houle et al., 2010). Plant phenomics has unfolded within an evolving niche to a blooming investigative platform. It can be defined as the multidimensional application of advanced tools and procedures applied for capturing the detailed data on plant growth, function, structure, and behavior in a given environment. It deals with the acquisition, organization, and evaluation of huge phenotypic data sets, and the design of intelligent models for the prediction of plant growth in multiple scenarios (Houle et al., 2010). Plant phenotyping is a crucial approach to study the relationship of plants with their environment, and can conduct at various grades of resolution from genome to the whole plant under diverse climatic conditions, from field to systematic controlled environments. However, for every level spotlight on specific traits, the final objective is to connect the information from bottom up to develop elite crop varieties (Walter et al., 2015; Araus et al., 2018). So, the plant phenomics tools are indispensable in modern breeding and provide an excellent way forward to develop next-generation crops.

### High-Throughput Phenotyping: A Step Toward Digital Agriculture

In recent years, state-of-the-art advanced phenotyping has emerged as a joint venture of multidisciplinary research groups to facilitate the launching of high-throughput phenotyping to expedite the next-generation breeding programs to ensure food security (Fasoula et al., 2020). High-throughput phenotyping allows high resolution imaging of thousands of plants for a better understanding of the insights of plant phenomics and genetics (Roitsch et al., 2019). It is the most promising technology that successfully incorporates plant science, engineering, math, information science, and computation with highly sophisticated tools of AI and ML to uncover diverse and intractable phenotypes of larger genotypes that are important to develop the best crop cultivars (Furbank et al., 2019). At present, several state-of-the-art phenotyping centers have been established in Europe, Australia, and USA as described in Table 5.

**Table 5:** List of major globally available high-throughput phenotyping facilities.

**Phenotyping system**	**Country**	**University/Institute/Collaboration**	**Weblink**
International Plant Phenotyping Network (IPPN)	Different partner countries	-	https://www.plant-phenotyping.org/
European Plant Phenotyping Network 2020 (EPPN)	Collaboration of 22 European countries	-	https://eppn2020.plant-phenotyping.eu/EPPN2020_start
North American Plant Phenotyping Network (NAPPN)	United States	-	http://nappn.plant-phenotyping.org/
WSU Plant Phenomics	United States	Washington State University	http://phenomics.cahnrs.wsu.edu/
Nebraska Innovation Campus (NIC)	United States	University of Nebraska–Lincoln	https://ard.unl.edu/phenotyping/nebraska-innovation-campus-greenhouse
Controlled Environment Phenotyping Facility (CEPF)	United States	Purdue University	https://ag.purdue.edu/cepf/
Plant Imaging Consortium (PIC)	United States	Arkansas State University	http://plantimaging.cast.uark.edu/
Center for Advanced Algal and Plant Phenotyping	United States	Michigan State University	https://prl.natsci.msu.edu/research-tech/center-for-advanced-algal-and-plant-phenotyping/
SLANTRANGE	United States	-	https://slantrange.com/company/
Austrian Plant Phenotyping Network (APPN)	Austria (Vienna BioCenter)	University of Innsbruck, University of Vienna, University of Natural Resources and Life Sciences	https://appn.at/plant-phenotyping-forum/
Australian Plant Phenomics Facility (APPF)	Australia	Australian National University, The University of Adelaide, Commonwealth Scientific and Industrial Research Organization (CSIRO)	https://www.plantphenomics.org.au/
McGill Plant Phenomics Platform (MP3)	Canada	McGill University	http://mustang.biol.mcgill.ca/mcgill_mp3_summary.html
Eastern Canadian Plant Phenotyping Platform (ECP3)	Canada	McGill University	https://www.mcgill.ca/macdonald/research/canada-foundation-innovation-grants/eastern-canadian-plant-phenotyping-platform-ecp3
Green Crop Network (GCN)	Canada	McGill University	https://www.greencropnetwork.com/
Biotron Experimental Climate Change Research Centre	Canada	The University of Western Ontario	https://www.uwo.ca/sci/research/biotron/
German Plant Phenotyping Network (DPPN)	Germany	Helmholtz Zentrum München	https://dppn.plant-phenotyping-network.de/
Jülich Plant Phenotyping Centre (JPPC)	Germany	Jülich Forschungszentrum	https://www.fz-juelich.de/ibg/ibg-2/EN/_organisation/JPPC/JPPC_node.html
Lemnatec	Germany	Bavarian State Research Center for Agriculture	https://www.lemnatec.com/
PhenomUK	United Kingdom	University of Nottingham	https://www.phenomuk.net/
Plant Growth Facility (PGF)	United Kingdom	Cranfield University	https://www.cranfield.ac.uk/facilities/plant-growth-facility
National Plant Phenomics Centre (NPPC)	United Kingdom	Aberystwyth University	https://www.plant-phenomics.ac.uk/
National Plant Phenotyping Infrastructure (NaPPI)	Finland	University of Helsinki and University of Eastern Finland	https://www.helsinki.fi/en/infrastructures/national-plant-phenotyping
Nordic Plant Phenotyping Network (NPPN)	Denmark	University of Copenhagen	https://nordicphenotyping.org/
Phenospex	Netherland	-	https://phenospex.com/products/plant-phenotyping/planteye- f500-multispectral-3d-laser-scanner/?gclid=CjwKCAjw-YT1 BRAFEiwAd2WRtgmWQ35a0QpZBB57eVxotF5zV7NlmYMjPQi6 COEOoRR-zQk6MVT4GhoCkUQQAvD_BwE
Netherlands Plant Eco-phenotyping Centre (NPEC)	Netherlands	Wageningen University & Research and Utrecht University	https://www.worldfoodinnovations.com/activities/facilities/netherlands-plant-eco-phenotyping-centre
Czech Plant Phenotyping Network (CZPPN)	Czech Republic	Palacký University Olomouc	http://www.czppn.com/
Phenome Networks	Israel	-	https://phenome-networks.com/es/
Weighing, Imaging & Watering Machines (WIWAM)	Belgium	-	https://www.wiwam.be/?gclid=CjwKCAjw-YT1BRAFEiwAd2WRtqXp5W2FVAAYMBGYTqM_oAonzekRfhxkX7ZmSK3MHWMBmjg4E-0aUxoC7DwQAvD_BwE
Tree Phenotyping Platform (TPP)	Sweden	Umea University	https://www.upsc.se/tree-phenotyping-platform-at-upsc.html
PHENOME- French Plant Phenotyping Network (FPPN)	France	INRA	https://www6.angers-nantes.inrae.fr/bia_eng/BIA-highlights/Major-projects/PHENOME

High-throughput plant phenomics operates within three approaches; firstly, the identification of a target trait regulating a unique stress response for precise, reproducible, accurate, and rapid data acquisition. The second and the third step based on a cutting-edge computer vision system enable the estimation and imaging examination of data and the computational analysis to forecast a biological response, respectively (Esposito et al., 2020). These hi-tech computer systems can lead current agriculture to digital agriculture, which uses AI and ML approaches to determine several factors like crop diseases, weeds identification, irrigation requirements, pesticide control, crop yield, and quality prediction (Zhang et al., 2017). There are numerous vital phenotypes that need to be investigated in order to dig out mysterious plant functions, or candidate phenotypes related to abiotic/biotic stresses and several agronomic traits. These multigenic traits must be spilt into constituent traits that can be observed, assessed, and evaluated *via* high-throughput advanced tools (Roitsch et al., 2019).

Tools like imaging, robotics, sensors, and software platforms have revolutionized the crop phenomics. The most common phenotyping platforms are unmanned aerial vehicles (UAVs), such as satellites, drones, phenotyping towers, environmental sensor networks, phenomobiles, autonomous ground vehicles, air crafts, helicopters, zeppelins, and field scanning platforms, which are currently used on a large scale (Liebisch et al., 2015). Non-invasive sensor-based phenotyping tools included; laser triangulation or red-green-blue (RGB) imaging system to determine the phenotypes of size, color, morphology, structure, texture, and growth of plant canopies. The hyperspectral and multispectral sensors can be used to measure moisture content, pigments composition, nitrogen content, and biophysical parameters. The thermal sensors were used for canopy temperature measurements to understand root physiology phenotypes. Light detection and ranging (LIDAR) provide the three-dimensional (3D) data for plant structural phenotypes (Araus et al., 2018).

Similarly, the automated image-based high-throughput phenotyping deals with the remote sensing and quantification of a large number of plant traits by capturing and analyzing the images at regular intervals with great accuracy (Jimenez-Berni et al., 2018). Image-based phenotyping is mainly non-destructive, enabling the desire traits to be assessed regularly during plant‘s life (Bao et al., 2019). At present, 3D imaging technology is getting attention in modern phenomics, including laser-based scanning and image-based approaches, which can produce 3D models to extract volumetric and spatial plant traits simultaneously (Paulus et al., 2014). For example, multiview stereo (MVS) is an excellent and a cost-effective 3D phenotyping platform for multiview imaging of plants at organ level (Hui et al., 2018). Nguyen et al. (2015) designed a 3D imaging system mounted with 10 digital high-resolution cameras supported by an illumination source to increase the surface imaging of plants. In another study, a 3D stereo-imaging unit was developed to visualize the traits related to plant height and rape seedling leaf area by installing two RGB cameras in an imaging chamber having a bright illuminous system (Xiong et al., 2017).

The emergence of field-based phenotyping provides a way forward to grab the full leverage of genetic gain and overcome the hurdles in breeding programs as it is the end-phenotypic expression of any genetic factor in relation to its environment (Singh et al., 2016; Araus et al., 2018; Bao et al., 2019). Field-based phenotyping platforms generally encompass ground wheeled, UAV, robotic-assisted system, tractor-driven, and cable-suspended phenotyping units, connected with high-performance sensors and cameras (Roitsch et al., 2019). These tools have been employed to estimate crop adaptability in natural conditions and can determine the canopy photosynthesis rate, leaf area index, plant height, performance, biomass, and disease symptoms (Jimenez-Berni et al., 2018). Recently, a cost-effective field-based high-throughput system MVS-Pheno is designed to monitor the shoot size of maize and offers exceptional ability to study large plant populations under diverse ecological zones (Wu et al., 2020). Similarly, a robotic field-based phenotyping system, which provide side-view stereo imaging, was developed to capture the plant height of sorghum at regular intervals of time (Bao et al., 2019).

### Pitfalls in Plant Phenomics

In modern breeding, genotype-to-phenotype is a major drawback, which hinders the advanced breeding programs mediated by high-throughput genomic and phenomic tools (Harfouche et al., 2019). The connection between genome-environment-phenotype offers an excellent understanding to study the high-throughput data indicating that the plant stress mechanism is far more challenging due to multidimensional impacts of environmental changes on phenotypic plasticity and eventually widening of the genotype–phenotype gap (Gosa et al., 2019). Currently, modern crop improvement platforms heavily rely on advanced GAB and meticulous assessment of plant traits to define experimental lines and map the desired genes (Yang et al., 2020). The integration of genomic data with high-throughput phenotypic data to extract biologically fruitful information is a key to success in modern breeding (Harfouche et al., 2019).

However, despite a recent progress in genomic tools, the phenotyping technology did not grow at a competing rate to integrate the phenotypic data with genomics. The failure to capture phenotypic data effectively has a major pitfall, which hampers crop improvement programs (Harfouche et al., 2019; Wu et al., 2020; Yang et al., 2020). Due to the lack of phenomics data, our ability to measure phenotypic traits lags behind the existing capability to draw genomic data. Hence, the bottleneck is moving from genomics to phenomics (Großkinsky et al., 2018). There are some critical bottlenecks in plant phenotyping like the less efficient assessment of captured trait data that could result in poor identification of candidate genes and to capture the allelic variations for traits. Phenotyping of germplasm required a control range of conditions in replicated trails, which was costly and labor intensive (Junker et al., 2015). Accurate phenotyping in natural environment is also a big pitfall in many crop breeding schemes due to the highly heterogeneous natural conditions. Additionally, traditional phenotyping technologies are laborious, subjective, tedious, often causing damage to plants, and keeping record end-point phenotype (Naik et al., 2017). Therefore, an ample advancement in plant phenomics is needed for crop improvement in the long run, which will improve screening ability, fast-tracking of the genetic gains, accurate scanning of plant health status, and filling the hole between genotypic and phenotypic variations (Wu et al., 2020).

## AI for Agriculture

In this digital world, AI is the most expeditiously rising technology in computer science and deals with the building of intelligent machines that mimic the intelligence of human mind (Harfouche et al., 2019). AI comprises ML algorithm models like deep neural network (DNN), artificial neural network (ANN), random forest (RF), support vector machine (SVM), and advanced hi-tech technology such as internet of things (IoT). AI is a mesmerizing hi-tech system with infinite applications in agriculture and opens up new horizons for digital agriculture (Montesinos-López et al., 2018). Systems are being designed to help the agricultural scientists for a better understanding of the plant behavior under diverse climatic conditions (Jeong et al., 2016). Recently, Summit, the world's most powerful supercomputer has been launched, which has the capacity to store 27,000 graphical processing units (GPUs) and unfolds an exciting way forward. AI can be a game changer and is pivotal for the next-generation crop revolution in the near future (Streich et al., 2020).

Recently, next-generation AI has gained significant attention in plant breeding to solve the problems related to abiotic/biotic stresses, herbicide resistance, crop yield, and soil fitness by developing the intelligent predictive models (Muraya et al., 2017). The applications of AI in agricultural production can be enormous because making AI-assisted spatial feature mining approaches can offer an exceptional prospect to incorporate the multi-omics results with high-throughput data sets captured *via* modern phenomics tools (Bolger et al., 2019). Al needs efficient and intelligent data mining that can assist breeders to accurately predict the agronomic factors and can also forecast crop performance under different conditions such as temperature, humidity, and soil type (Großkinsky et al., 2018; Harfouche et al., 2019).

Jiang et al. (2004) have developed more precise yield predicting models and projected wheat yield using multiple linear regression (MLR) and ANN models with satellite-assisted climate and vegetation indices in North China. An UAV-mediated phenotyping system aided by multispectral imaging and AI was performed to estimate the phenotypic features on field crops (Ampatzidis and Partel, 2019). Hemming et al. (2019) carried out AI to successfully control a greenhouse as compared to manually controlled greenhouse for vegetable production.

### ML and Big Data Analytics

Machine learning is an emerging and a promising application of AI, which can be defined as the state-of-the-art computer-based systems that make the machine more intelligent to learn automatically and improve its ability without being stringently computed (Singh et al., 2016). Advanced ML algorithms have revealed a great potential in making highly precise and efficient pipelines for data analysis to enhance the breeding performance and ultimately crop productivity (Singh et al., 2018). For an accurate trait detection, several ML approaches have been employed, which can be divided into supervised/unsupervised and generative/discriminative learning model (Ghosal et al., 2018). In addition, many easily available programming languages or packages like MATLAB, ImageJ, and Python have been made to support or execute computer-based image pre-processing (Schindelin et al., 2012). ML tools can be applied to breakdown the multimodel data and identify the plant stresses and examine plant-pathogen association and interaction of other stresses with plants (Singh et al., 2018). One of the main benefits of ML tools for plant biologists is to get a chance to discover data sets in order to explore the patterns by analyzing the multitrait simultaneously (Shakoor et al., 2017).

Recently, an economical and a high resolving power image-based phenotyping system coupled with ML was designed to capture the root images of hundreds of plants to study the root system architecture traits in soybean (Falk et al., 2020). Likewise, root phenotyping of mature plants was performed *via* ML algorithms (RFs and SVM) to screen the most distinguishing root traits (Zhao et al., 2016). Additionally, sensor tools with ML algorithms can be applied to forecast crop yield under field conditions (Pantazi et al., 2016) and the plant growth trends for future predictions (Lee et al., 2018). An accurate ML model was developed to predict the photosynthesis activity in crops, and the result showed that the spectra-based phenotyping technique has the ability to improve photosynthesis capacity (Heckmann et al., 2017). ML has been employed for the early detection of various plant diseases. For example, ANNs and RFs tools were used to envisage the risk assessment of wheat *Stagonospora nodorum blotch* (Mehra et al., 2016). A realtime pipeline for phenotyping using ML was established to estimate the severity of abiotic and biotic stress in soybean. This system can be assisted to enhance the genetic gains by allowing the automatic stress trait identification and stress scouting applications (Naik et al., 2017).

### ML-Assisted GS

Genomic selection enables the quick screening of elite germplasm and expedites the crop breeding cycle (Crossa et al., 2017). Currently, GS depends on innovations in ML tools and retrieval of large genotyping data sets related to agronomically important phenotypic traits for genomic prediction (Tong and Nikoloski, 2021). So, GS models are the core elements of ML that intend to design and examine the model performance through an array of training data. In GS models, the genotyping data such as SNPs are the input, and the predicted phenotypic trait is the final output. The leverage of exploiting ML, especially DL to analyze the GS is that it may obtain highly complex interactions and deliver greater predictability (González-Camacho et al., 2012). There are several examples of DL application for GS in crop improvement programs because CNNs are very accurate in predicting phenotypic traits (Pérez-Enciso and Zingaretti, 2019).

Deep learning techniques have been used in a multitrait situation, for example, wheat population exhibited that the prediction of multitrait DL is the same as a single-trait model, but somewhat superior when analyzing in relation to univariate DL models (Montesinos-López et al., 2019a,b). González-Camacho et al. (2016) demonstrated that the probabilistic neural network (PNN) is a promising strategy for GS in crop breeding by testing the wheat and maize population to predict the genotypes related to good or bad groups. The results showed that PNN models are more efficient than multilayer perceptron (MLP) models. Liu et al. (2019) study soybean to predict five traits employing CNN models and revealed better performance. A CNN-based DeepGS method was developed to study the grain-related yield in wheat population (Ma et al., 2018).

## Speed Breeding

The world is attracted by the most fascinating technology of speed breeding. The scientist from the University of Queensland inspired by the NASA to grow the wheat plants in space under artificial lights. Watson et al. (2018) successfully developed the protocols for different plant species under a speed breeding system. Speed breeding is a powerful strategy to shorten the crop generation time and expedite the breeding programs for crop improvement (Watson et al., 2018). Speed breeding mimics daily dawn and dusk, and plants are subjected to an extended photoperiod of about 22 h by using a combination of different light sources. It provides an extended day length with optimal light intensity coupled with controlled temperature to increase the photosynthesis activity, which results in quick flowering and early seed development to reduce generation time (Ghosh et al., 2018).

Speed breeding is revolutionizing the agriculture and can be executed to accelerate the crop breeding activities such as crossing, back crossing, rapid gene identification, mapping population, pyramiding of traits, and developing transgenic pipelines (Hickey et al., 2019). In conventional breeding, only 1–2 generations per season of any crop can be achieved but in speed breeding up to four generations of *B. napus* and six generations of *Hordeum vulgare, Triticum aestivum, Pisum sativum, Cicer arietinum*, and *B. distachyon* (Watson et al., 2018). Furthermore, it can provide a robust, an efficient, and an economical platform to carry out the crop improvement project in an integrated way from genomics to phenomics. It may include the candidate genes discovery through GAB approaches such as pan-genome assembly, GS, and GBS to multiplex gene editing, or metabolic pathway editing for desired traits followed by high-throughput phenotyping to visualize the results. The integration of speed breeding with next-generation metabolomic tools can also be used for a rapid risk assessment of gene-edited crops in multiple generations in a robust manner (Razzaq et al., 2019a). Hence, speed breeding technology will offer an exciting way forward for crop improvement by integrating it with next-generation OMICS tools to accelerate the crop breeding programs.

## Outlook

Human population explosion and adverse climatic changes are posing extreme challenges for promising global food security. Next-generation breeding technologies offer a robust platform to develop high yielding and climate-resilient crops. GAB has made a significant impact on plant research, and ground-breaking pan-genome techniques permit to capture the full landscape of genetic variations. Hence, we are expecting a genomic data explosion as third-generation sequencing combined with the pan-genome concept will possibly construct large gene repertoires of landraces, cultivated plants, and CWR. The development of super-pangenomes, a combination of different pan-genomes, depicts the whole genetic profile of any specific genus and will help for pangenome-led haplotype breeding for crop improvement. The assembly of plant pan-omes will allow to study the genetic variation in single cell level and will provide huge data sets from genome to phenome for a quick and an accurate phenotypic identification. The integration of pan-omes with speed breeding and genome editing will accelerate the trait-specific breeding. Applications of AI such as DL in super pangenomes construction will help to retrieve huge data sets. The potential of DL in combining the sequencing data will enable to construct the models for GS and trait prediction.

Next-generation CRISPR systems, like *de novo* domestication, tissue-specific editing, prime editing, and fine tuning of gene expression, are now giving plant scientists an unprecedented prospect to manipulate the plant genomes with more accuracy and precision. The potential of an CRISPR/Cas system to edit the plant organelles like chloroplast and mitochondria is yet to be explored because of the unavailability of delivery vectors that can enter into these organelles. In the near future, chloroplast and mitochondria can be targeted by transferring multiple gRNAs to achieve cell-based editing. Also, some breakthroughs like the delivery of CRISPR/Cas machinery being the biggest drawback in genome editing are still needed. The RNP or carbon nanotubes- (CNTs-) based delivery system is transferred directly into inflorescence tissues, pollen grains, and apical meristems, or it is sprayed to already grown plant to get express edits in the field or under speed breeding chambers (Hickey et al., 2019). Performing the genome editing under the speed breeding condition will provide a controlled system to speed up the crop breeding cycles with a minimum cost and will be a technological breakthrough to get non-transgenic plants without tissue culturing. It will tag as non-GMOs with more public acceptance and avoid the strict regulatory affairs.

The exciting phenotyping platforms inspired by AI, like robotics, 3D imaging sensors, and their integration with other OMICS date, are taking plant science to new heights. With multiscale, multidimensional, and multidomain phenomics knowledge, we promptly require the next-generation approaches of swarm intelligence, hybrid intelligence, AI, data fusion, and DL to establish big data handling methods.

**Figure 7:**
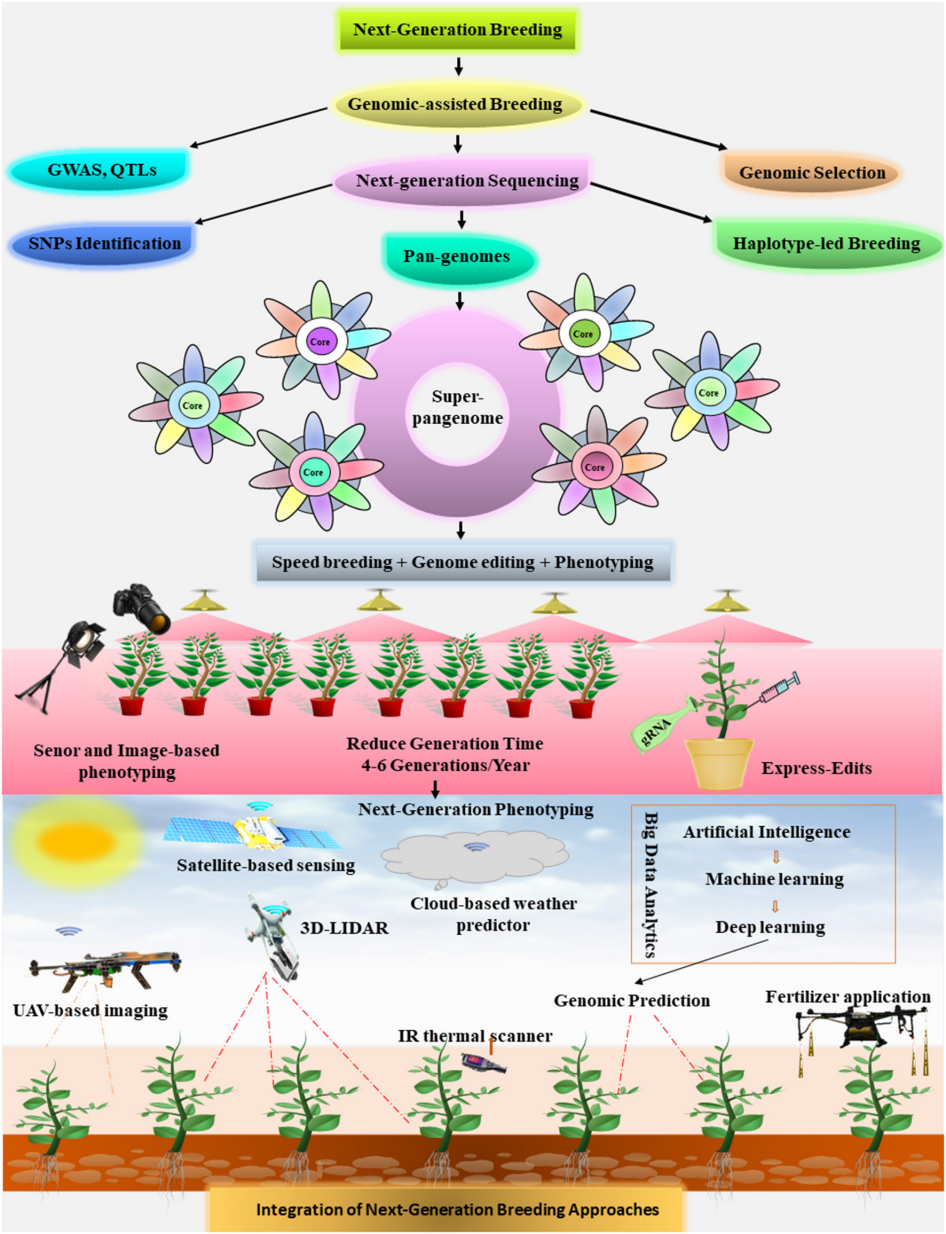
Integration of next-generation breeding pipelines for crop improvement.

To conclude, a powerful unified strategy is needed to tackle the current challenges by integrating the multidisciplinary next-generation strategies and scientists to work under one umbrella and carry out mega projects to boost up the crop production in a rapid way as proposed in Figure 7.

The multidisciplinary tools from genomics to phenomics should be carried out in a well-integrated manner to overcome the current hurdles in plant research. By employing the integrated next-generation approaches, future crop breeding can attain irreversible success to ensure food security and will fulfill the food demands of rapidly increasing population in decades to come.

## Author Contributions

AR conceived the idea. AR and FS led the writing of manuscript. NA helped in revising the manuscript. PK and SW reviewed, edited, and improved the manuscript. All authors contributed to the article and approved the submitted version.

## Conflict of Interest

The authors declare that the research was conducted in the absence of any commercial or financial relationships that could be construed as a potential conflict of interest.

## Footnotes

^1^FAOSTAT (2020). Available: http://www.fao.org/faostat/en/#data (accessed July 30, 2020).

^2^EM-DAT (2020). Available: https://www.emdat.be/ (accessed March 29, 2020).
